# Predicting Cell Types and Genetic Variations Contributing to Disease by Combining GWAS and Epigenetic Data

**DOI:** 10.1371/journal.pone.0054359

**Published:** 2013-01-30

**Authors:** Anna Gerasimova, Lukas Chavez, Bin Li, Gregory Seumois, Jason Greenbaum, Anjana Rao, Pandurangan Vijayanand, Bjoern Peters

**Affiliations:** La Jolla Institute for Allergy and Immunology, La Jolla, California, United States of America; Harvard Medical School, United States of America

## Abstract

Genome-wide association studies (GWASs) identify single nucleotide polymorphisms (SNPs) that are enriched in individuals suffering from a given disease. Most disease-associated SNPs fall into non-coding regions, so that it is not straightforward to infer phenotype or function; moreover, many SNPs are in tight genetic linkage, so that a SNP identified as associated with a particular disease may not itself be causal, but rather signify the presence of a linked SNP that is functionally relevant to disease pathogenesis. Here, we present an analysis method that takes advantage of the recent rapid accumulation of epigenomics data to address these problems for some SNPs. Using asthma as a prototypic example; we show that non-coding disease-associated SNPs are enriched in genomic regions that function as regulators of transcription, such as enhancers and promoters. Identifying enhancers based on the presence of the histone modification marks such as H3K4me1 in different cell types, we show that the location of enhancers is highly cell-type specific. We use these findings to predict which SNPs are likely to be directly contributing to disease based on their presence in regulatory regions, and in which cell types their effect is expected to be detectable. Moreover, we can also predict which cell types contribute to a disease based on overlap of the disease-associated SNPs with the locations of enhancers present in a given cell type. Finally, we suggest that it will be possible to re-analyze GWAS studies with much higher power by limiting the SNPs considered to those in coding or regulatory regions of cell types relevant to a given disease.

## Introduction

Asthma is a chronic inflammatory disease, characterized by reversible airway obstruction and increased bronchial hyperresponsiveness. This complex disorder is influenced by the interdependencies between various factors - genetic and environmental being the most important ones. The estimate that 35–80% of the variation in the risk of asthma can be attributed to genetic variation has spurred a number of genome-wide association studies (GWASs) of asthma [1]–[17], making it one of the best studied diseases to date. Most of these studies involved genotyping asthmatic vs. non-asthmatic donors using single nucleotide polymorphism (SNP) arrays that can detect the presence or absence of up to a million SNPs [10]. Using information from studies such as The 1000 genomes [18] and Hap Map projects [19], the presence of additional SNPs not present on the genotyping arrays can be extrapolated by imputation [20], [21]. GWASs have led to the discovery of a large set of SNPs that are significantly more frequent in patients with asthma compared to individuals without asthma or healthy controls. However, identifying a functional link between the presence of an asthma-risk SNP and development of disease has not been straightforward [22], primarily because the majority of identified SNPs are located in non-coding regions so that there is no obvious expected phenotype or function, but also because of the tight genetic linkage of SNPs in a haploblock, only few of which will have a functional effect.

Recently, there has been a vast acceleration in the identification of non-coding genomic elements that regulate gene transcription [23], [24]. This has been enabled by the advent of genome-wide chromatin analysis, such as profiling of the histone mark H3K4me1 (histone 3 lysine 4, monomethylation). This mark is found on histones associated with genomic regions accessible to regulatory DNA-binding proteins (transcription factors), which thereby function as enhancers of transcription. Enhancers could also be marked by other protein modifications, e.g. H3K4me2 (histone 3 lysine 4, dimethylation), H3K27ac (histone 3 lysine 27, acetylation), as well as histone variant H2A.Z [25]–[27]. In this study, we focused on the H3K4me1 and H3K27ac marks as more data were publicly available for these markers.

Notably, the location of enhancers is highly cell-type specific [28], supporting the notion that different cell types maintain their specialized functions by selectively activating different regulatory regions of the genome [29]. Moreover, several recent studies have shown that disease-associated SNPs are enriched in enhancers [29]–[32].

Based on these considerations, we tested the hypothesis that SNPs associated with a specific disease are more frequently found in enhancers specific to cells that are relevant for the disease. Using asthma as an example, we document a significant enrichment of asthma-associated SNPs in genomic regions marked by H3K4me1 in CD4+ T cells, which are known to contribute to asthma pathogenesis. In contrast, cells from brain, breast and skeletal muscle tissues unrelated to asthma, are depleted of asthma-associated SNPs in their enhancer regions. Thus, the methodology we report here can be utilized to make an unbiased prediction of which cell types contribute to disease pathogenesis, and which disease-associated SNPs are likely to be functionally important.

## Results

### The Majority of Asthma-associated SNPs is Located in Non-coding Regions

**Figure 1:**
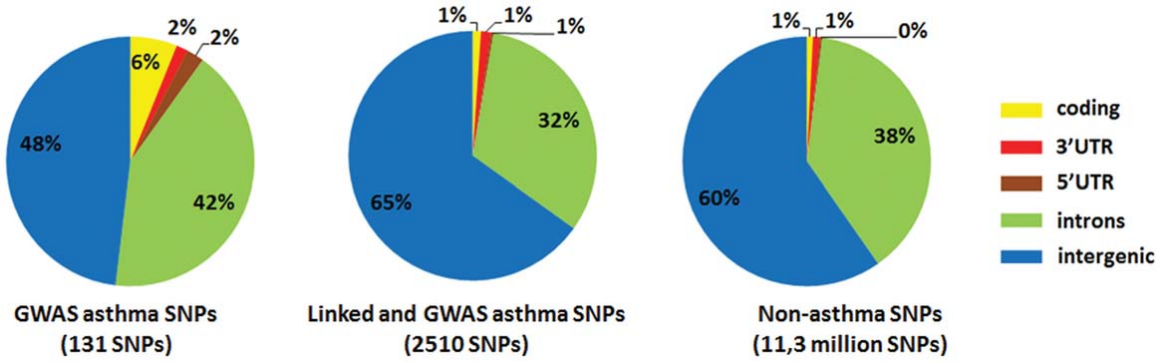
Distribution of the SNPs in coding, 5′-UTR, 3′-UTR, introns and intergenic regions. Three sets of SNPs are shown. SNPs identified as being significantly associated with asthma according to the GWAS Integrator database [33] are shown on the left. The middle shows the same set of SNPs extended by those that are in tight genetic linkage (r^2^ = 0.8) according to HaploReg [30]. On the right, the distribution of common SNPs that are not associated with asthma is shown. The distribution of SNPs into coding, 5′-UTR, 3′-UTR, introns, and intergenic regions was done using RefSeq datasets from the UCSC Genome Browser [34], [35].

We retrieved all known asthma-associated SNPs from the GWAS integrator database [33], resulting in 131 SNPs (Table S1 in File S1). For all these directly disease-associated SNPs, we also retrieved SNPs in tight genetic linkage (n = 2510, r^2^ = 0.8) based on the most recent release of HaploReg [30] (Table S2 in File S1). As a background control set, we assembled non-asthma-associated SNPs from DbSNP135Common dataset of the UCSC Browser [34], [35]. The distribution of the three sets of SNPs in coding regions (cds), 5′-untranslated regions (5′-UTRs), 3′-UTRs, introns, and intergenic regions is shown in Figure 1. As expected, asthma-associated SNPs were highly enriched in coding sequences compared to the background set of SNPs, and a significant but lower enrichment was found for 3′- and 5′-UTRs. However, the largest proportion of asthma-associated SNPs was found in introns and intergenic regions, i.e. non-coding sequences known to contain enhancers.

### Non-coding SNPs are Significantly Enriched in Enhancers

In an elegant study by the Kellis group [36], genomic regions in CD4+ T cells were comprehensively classified into different chromatin states using a Hidden Markov Model. We used these classifications to examine if any chromatin states were enriched for asthma-associated SNPs. As shown in Table S3 in File S1, we found enrichment of disease-associated SNPs in promoter states (state 1–9, and 11), and in some states associated with transcription (states 12, 14, 21 and 24) - result already anticipated from Figure 1. In addition, we have detected an enrichment of asthma SNPs in many active intergenic states (State 29, 30, 34–36).

In contrast, regions with heterochromatin state and repetitive elements (states 40–42, and 48) showed high depletion for disease-associated SNPs, as did some states associated with transcription (states 15, 17–19, 23 and 28).

Notably, we also found significant enrichment in States 9, and 34, which are associated with promoter and enhancer regions, suggesting that SNPs present in non-coding regions (enhancers and promoters) contribute to disease pathogenesis by perturbing the transcriptional regulation of an associated gene.

### The Location of Active Enhancers is Primarily a Function of Cell Type

**Figure 2:**
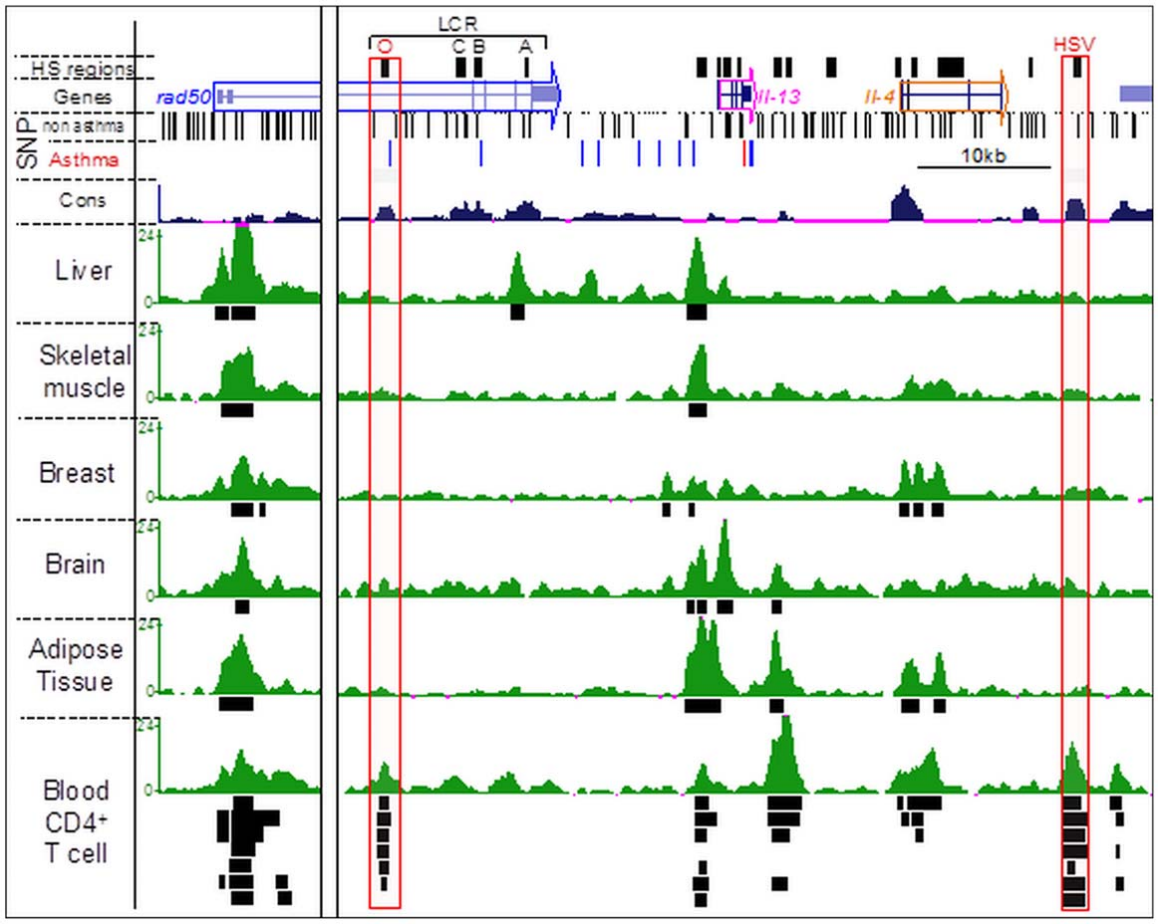
Asthma-associated SNPs and H3K4me1-enriched regions (enhancers) in the human Th2 cytokine locus of different cells and tissue types. From top to bottom, using the UCSC genome browser, are displayed: the conserved DNAse hypersensitivity regions identified in mouse T cells (HS regions), the gene track (genes), all the SNPs not associated with asthma, the SNPs associated with asthma, the species conservation track, the H3K4me1 ChIP-seq track (green) for the different cell and tissue types (named on the left) underlined by corresponding peak calling track (black boxes). For the blood CD4+ T cells, peak calling tracks from seven samples/cell-types are displayed. The red boxes show H3K4me1 peaks that are present only in CD4+ T cells (LCRO and HSV).

Enhancers have previously been reported to be tissue and cell-type specific [37], [38]. To determine if the SNP enrichment in enhancers that we found for CD4+ T cells (which are known contributors to asthma) was a cell-type specific effect, we compared the available data on the distribution of the H3K4me1 modification for human CD4+ T cells and several other human tissues/organs using published studies. We obtained all available H3K4me1 Chromain ImmunoPrecipitation (ChIP)-Seq data from the epigenome atlas [39], [40] limiting our analysis to data for which both IP and control input samples were available. A total of 37 samples from 19 distinct cell types and 8 tissues were retrieved, and putative enhancers in each dataset were identified by H3K4me1 peak calling (Materials and Methods). As an example of cell type-specific distribution of H3K4me1, Figure 2 shows the extended Th2 cytokine locus. The well-known locus control region LCR-O and the hypersensitivity site (HS) V, which both function as enhancers in CD4+ T cells, are marked in red boxes [41]. Importantly, there are H3K4me1 peaks at these regions in six CD4+ T cell types (Figure 2), but no peaks were found in the kidney, liver or brain cells. This result demonstrates that our method for calling H3K4me1 peaks accurately identifies known enhancers.

**Figure 3:**
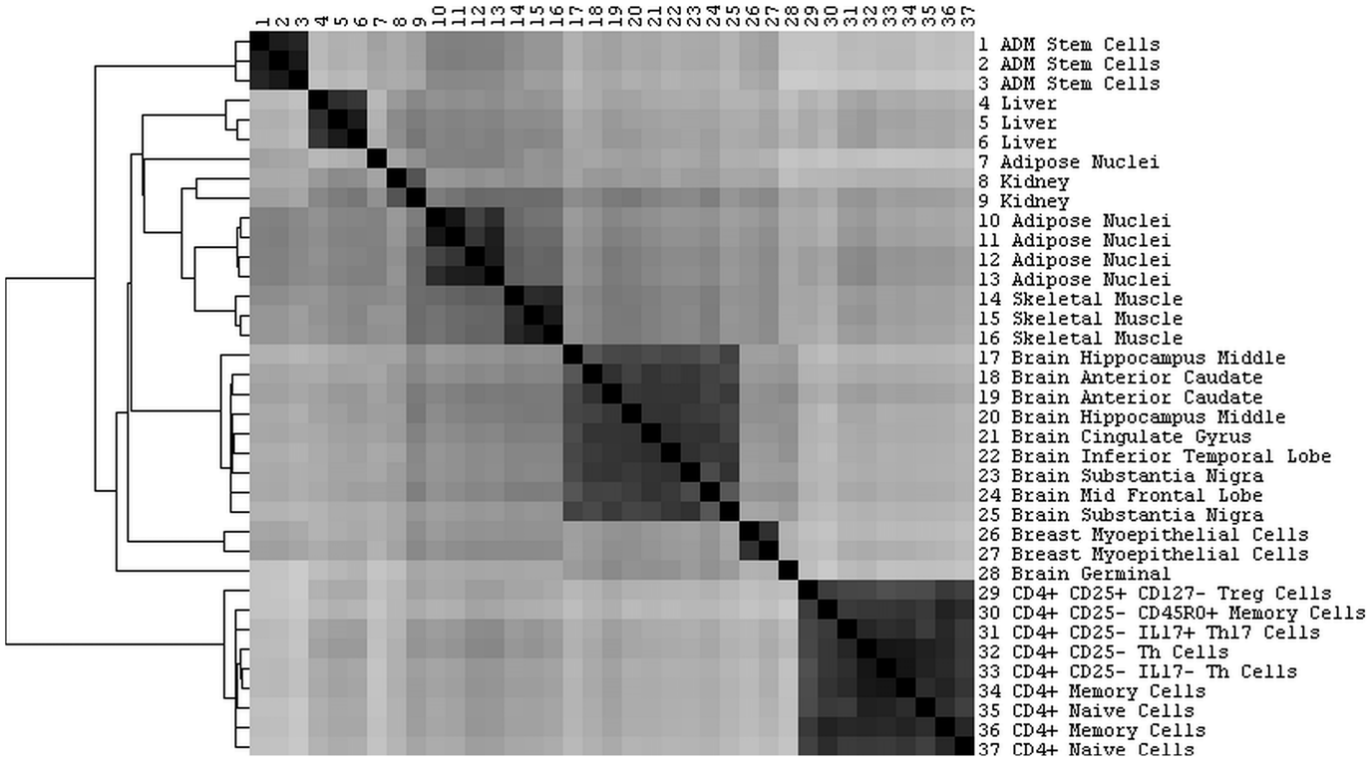
The location of enhancers is cell-type specific. The plot depicts pairwise comparisons of the location of enhancers in different datasets using Matthew Correlation Coefficients (MCC). Black indicates a high correlation between enhancers in two cell types. The 37 studied datasets form distinct clusters that correspond to different cell- or tissue types.

Next, we compared the enhancers identified in different datasets by calculating pair-wise Matthews correlation coefficients (MCC), which quantify the overlap of enhancers on a per nucleotide basis (see Materials and Methods). Figure 3 depicts a MCC heatmap for all studied datasets, which fell into 10 distinct clusters using a cutoff for pairwise MCC values above 0.55. Two samples formed isolated clusters, and were considered outliers (sample 7, Adipose Nuclei, and sample 28, Brain Germinal). Apart from these exceptions, all datasets from the same cell types but different laboratories or different donors fell into the same cluster. For example, all nine datasets of CD4+ T cell-types were in one cluster, as were all dataset from different brain regions. This demonstrated that the genomic location of enhancers marked by H3K4me1 was primarily determined by cell type, and donor-to-donor variability was not a significant factor.

### Non-coding SNPs Associated with Asthma are Enriched in CD4+ T Cell Specific Enhancers

To determine if there was differential enrichment of asthma-associated SNPs in the enhancers of different cell types, we merged the enhancers identified in each of the eight cell/tissue type specific clusters displayed in Figure 3 to obtain a single set of enhancers per cell/tissue type. In each of these eight sets, we determined how many asthma-associated SNPs were found in enhancers, and calculated enrichment over the background SNP distribution. As shown in Table 1, we saw the biggest enrichment (2.11) of asthma-associated SNPs in CD4+ T cells, and we also detected some enrichment of asthma SNPs in liver, adipose nuclei and adipose stem cells. However, for cells from skeletal muscle, breast myoepithelial or brain, asthma-associated SNPs were depleted in enhancer regions. This depletion makes intuitive sense, as these cell types are thought not to be major contributors to asthma. As a control, we also calculated the enrichment of asthma SNPs using different background distribution, namely the SNP panels present on the most commonly used typing arrays (Affymetrix 6.0, Illumina 550, Illumina 650 and Illumina 1MDuo). As was expected, we consistently found the highest enrichment of asthma SNPs in enhancers of CD4+ T cells, and also determined enrichment in four tissues considered significant in our original analysis (CD4+ T, liver, adipose nuclei, and adipose derived mesenchymal stem cells), while finding lesser enrichment in the remaining tissues (kidney, brain, skeletal muscle and breast myoepitethelial cells, Table S4 in File S1).

**Table 1:** Asthma SNPs enrichment in enhancers of different tissues.

Cell type	asthma SNPs (out of 2,510)	non-asthma SNPs (out of 11.3 million)	enrichment	p-value for chi-square test
CD4+ T Cells	430	921,179	2.11	<0.0001
Liver	282	970,466	1.31	<0.0001
Adipose Nuclei	376	1,402,437	1.21	<0.0001
Adipose Stem Cells	378	1,478,423	1.15	0.0028
Kidney	177	755,135	1.06	0.4386
Breast Myoepithelial Cells	182	978,214	0.84	0.0135
Skeletal Muscle	192	1,047,059	0.83	0.0058
Brain	202	1,190,305	0.77	<0.0001

Next, we performed a similar analysis using a second enhancer marker, H3K27ac (Table 2). H3K27ac is known to distinguish active enhancers from inactive/poised enhancer elements containing H3K4me1 alone [38]. Due to the more selective nature of this marker, we expected to find a higher enrichment of disease associated SNPs. However, we found that asthma- SNPs enrichment in CD4+ T cell H3K27ac peaks was essentially the same as in CD4+ T cell H3K4me1 peaks. (2.12 and 2.11 respectively). Additionally, almost all asthma SNPs that belong to CD4+ T cell H3K27ac peaks were also located in CD4+ T cell H3K4me1 peaks (230 out of 254). Notably, by focusing on H3K27ac mark we not only fail to obtain higher enrichment of disease associated SNPs, but we also lose about 40% of possible functionally important SNPs. Overall, the analysis if H3K27ac marks supports the findings we had for H3K4me1, and suggests that an analysis of a single marker H3K4me1 might be sufficient to obtain all information.

**Table 2:** Asthma SNPs enrichment in H3K27Ac of different tissues.

Cell type	asthma SNPs(out of 2,510)	non-asthma SNPs (out of 11.3 million)	enrichment	p-value for chi-square test
CD4+ T Cells	254	539,868	2.12	<0.0001
Adipose Nuclei (1 sample)	155	478,155	1.46	<0.0001
Skeletal Muscle (1 sample)	128	499,663	1.16	0.0931
Brain (1 sample)	69	519,313	0.6	<0.0001

**Figure 4:**
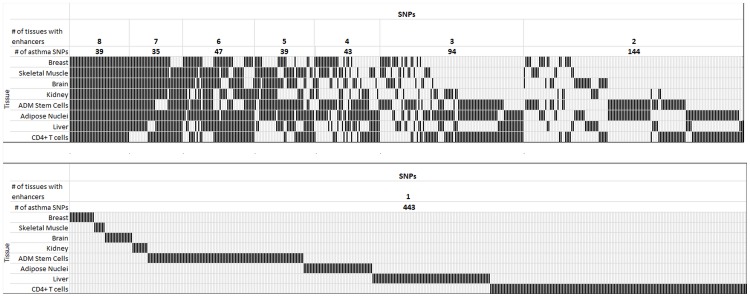
Distribution of enhancers in asthma-associated SNPs for different cell types. For each SNP and cell type, a black bar indicates that an enhancer is overlapping the SNP in that cell type. Cell types are ordered by their enrichment for asthma-associated SNPs in enhancers from breast tissue with low enrichment at the top to CD4+ T cells with high enrichment at the bottom. SNPs are ordered by how commonly they overlap with enhancers in different cell types from those with enhancers present in all 8 cell types on the left to with enhancers in just 1 cell type on the right. Asthma-associated SNPs with no enhancer in any cell type are left out from the graph.

**Figure 5:**
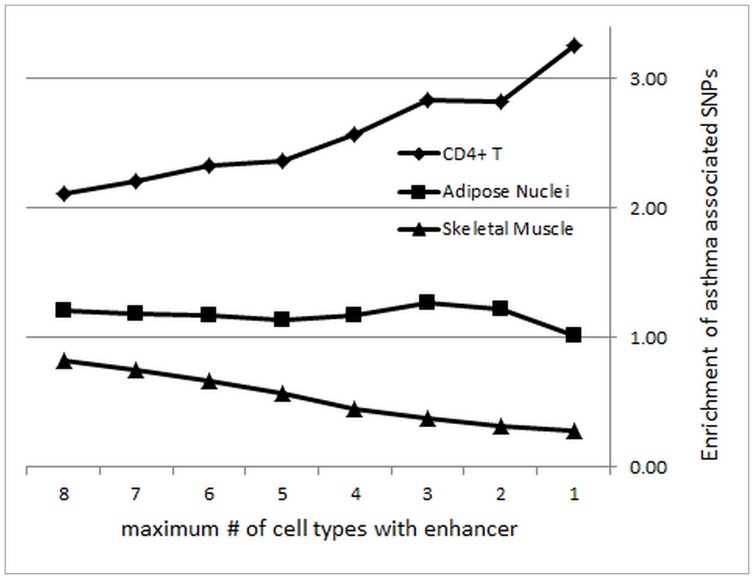
Distribution of enhancers in asthma-associated SNPs for different cell types. Plotted is the enrichment of asthma-associated SNPs compared to background SNPs in genomic regions in which there are CD4+ T enhancers, and anywhere from –0 to 7 additional cell types that also have a peak in that region.

We asked whether the enrichment for asthma-associated SNPs could be increased by excluding ubiquitous enhancers found in many cell types/tissues. Figure 4 shows for each tissue the number of asthma-associated SNPs located in cell type-specific enhancers (black boxes show asthma-associated SNPs in which an enhancer is present in that cell type whereas open boxes show SNPs with no enhancer). Of the 884 SNPs that fell into enhancers in one or more of the eight tissues, the by far largest number (443) was found exclusively in a single tissue. A much smaller number (39) was found in all eight tissues. Figure 5 shows that the enrichment of asthma-associated SNPs compared to background SNPs in enhancers of CD4+ T cells increased moderately when enhancers found in many other cell types were excluded.

The highest enrichment was determined by considering either the SNPs unique to CD4+ T cells, or SNPs found in up to two other cell types. By contrast, enhancers in skeletal muscle cells were clearly depleted of asthma-associated SNPs, and such depletion became increasingly pronounced when excluding enhancers also found in other cell types. A similar, but less pronounced, trend was found for cells from breast and brain tissues (Table S5 in File S1), whose enhancers are depleted of asthma-associated SNPs and become more depleted when eliminating enhancers shared with other cell types. For the remaining cell types (liver and adipose tissues), which showed an enrichment of asthma SNPs in their enhancers, the enrichment increased further when considering subsets of enhancers unique to these cells.

**Figure 6:**
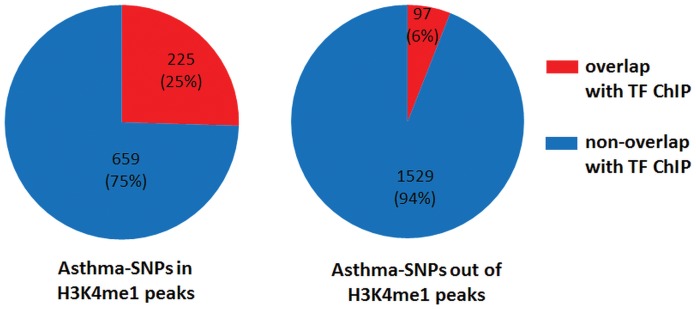
Distribution of the asthma-associated SNPs in TFBSs. Two sets of asthma-associated SNPs are shown. Asthma-associated SNPs that belong to any of H3K4me1 peaks called in this study are shown on the left. On the right, the distribution of asthma-associated SNPs that are located in any of H3K4me1 peaks. The distribution of SNPs into overlapping and non-overlapping TFBSs was done using TFBSs by ChIP-seq dataset from the ENCODE (Release 2) [55]. The TFBSs ChIP-seq data were obtained from UCSC Genome Browser [34], [35].

To examine the putative functional mechanism of asthma SNPs in enhancers, we were interested if might modulate in Transcription Factor Binding Sites (TFBSs). Figure 6 shows the distribution of asthma-associated SNPs in TFBSs determined by ChIP-seq experiments. Strikingly, asthma-associated SNPs located in enhancers overlap with TFBSs four times more often than those not located in enhancers. This finding supports our hypothesis that non-coding disease-associated SNPs in enhancers are more likely to be functional, and may do so by disrupting binding of transcription factors.

In summary, our analysis demonstrates that focusing on enhancers present in a few cell types further enriched asthma-associated SNPs compared to background SNPs. Since by far the greatest enrichment was observed at enhancers of CD4+ T cells which are known to contribute to asthma, our approach is capable of simultaneously prioritizing those SNPs that are more likely to contribute to a given disease, and identifying the cell types involved.

### Prediction of Putative Functionally Important SNPs

**Figure 7:**
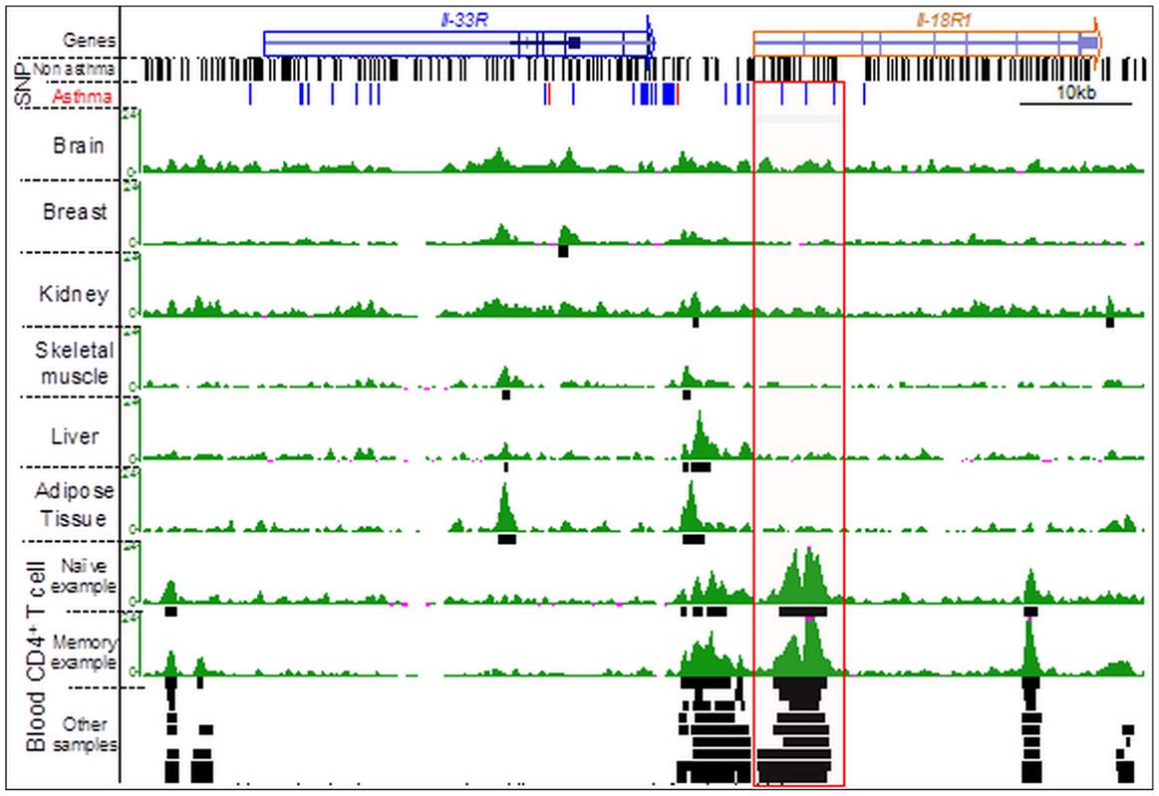
Asthma-associated SNPs and H3K4me1 (enhancer) enriched regions in the human IL-33R locus of different cell/tissue types. From top to bottom, using the UCSC genome browser, are displayed: the gene track (genes), all the SNPs not associated with asthma, the SNPs associated with asthma (red are GWAS-identified SNPs, blue are SNPs in linkage disequilibrium), H3K4me1 ChIP-seq track (green) for different cell/tissue types (named on the left) underlined by the corresponding peak-calling track (black boxes). For the blood CD4+ T cells, peak calling tracks from seven samples/cell-types are displayed. The red box shows an H3K4me1 peak that is present only in CD4+ T cells.

Based on the reasoning that functional disease-associated non-coding SNPs are likely to dysregulate gene expression by disrupting function of key *cis*-regulatory elements (enhancers) present in cell types that drive disease pathogenesis such as CD4+ T cells, we predict that SNPs present in such cell-type-specific enhancer regions (marked as red dotted line, Figure 2) are more likely to be biologically important than SNPs present in common enhancer regions or in non-enhancer regions. Thus we speculate that the non-coding SNPs (red boxes) present in the LCR-O and HS-V enhancers of the Th2 cytokine locus (Figure 2), the 5′ end of the *IL18R1* gene in the *IL1RL1*/*IL18R1* locus (Figure 7), and *IKZF3* locus (Figure S1 in File S1) are likely to modulate the function of the corresponding cell-type-specific enhancers in CD4+ T cells. Therefore, this enhancer and the risk-SNPs it bears are likely to the most relevant to asthma pathogenesis and experimental studies that seek to validate function of disease-associated SNPs should prioritize these.

## Discussion

Unraveling how common genetic variations contribute to disease development is complicated, as the effect of a genetic variation may be limited to a certain developmental stage and/or cell type, and may be dependent on the presence of additional environmental factors. Our analysis of asthma-associated SNPs demonstrates how this problem can be tackled by the use of epigenetic information, which identifies which genomic regions are active in different cell types.

Previously, Pham and colleagues explored the association between potential enhancers and disease-associated variants extracted from a comprehensive GWAS catalogue [42]. Their primary focus was on promoter-distal regions marked by H3K4me1 and/or H3K27ac. Specifically, the authors have validated a novel macrophage-specific enhancer signature encompassing ETS, CEBP, bZIP, EGR, E-Box and NFkB motifs by ChIP-sequencing, which confirmed their associations with epigenetic changes related to differentiation. Another, recent study by Maurano et al. [43] examined the distribution of 5,654 noncoding significant associations (5,134 SNPs) for 207 diseases and 447 quantitative traits. By combining with the deep genome-scale maps of regulatory DNA marked by DNase I hypersensitive sites (DHSs), their study has revealed a collective 40% enrichment of GWAS SNPs in DHSs. For externally replicated non-coding SNPs, 69.8% reside within a DHS. Of GWAS SNPs in DHSs, 93.2% (2,874) overlap a transcription factor recognition site. Common variants associated with specific diseases or trait classes were systematically enriched in the recognition sequences of transcription factors governing physiological processes relevant to the same classes. Our studies are in agreement with these findings, and suggest that the combined analysis of GWAS and epigenetic information predicts which SNPs are more likely to be functionally contributing to disease and in which cell types these effects will be noticeable**.** With the rapidly increasing amount of epigenetic data for different human tissues in different developmental stages, such analysis will become increasingly powerful.

Significant progress has been made with respect to the ENCyclopedia Of DNA Elements (ENCODE) project after our initial results were submitted for publication [44]–[45]. Multiple types of ENCODE data can now be linked with disease-associated SNPs, that could help pinpointing regulatory regions with significant enrichment for functional SNPs [46]. Researchers have employed ENCODE epigenetic data as a guide to unveil regulatory regions in which genetic variants could affect a given complex trait. For instance, Farrell et al. [47] applied ENCODE data to uncover the function of a DNA fragment encompassing a 3-bp deletion polymorphism, which is shown to have enhancer-like activity. The 3-bp deletion polymorphism could possibly represent the most significant functional motif accounting for HBS1L-MYB intergenic polymorphism associated with the trait of interest, fetal hemoglobin.

Our present analysis is meant more as a proof of concept than an optimized, definitive study; there are multiple ways in which it can be significantly improved. First, the amount of published asthma-associated SNPs is continuously increasing, with a gain of 65% from January to October 2012, and additional SNPs will continue to be discovered. Moreover, rather than relying on SNPs that reach statistical significance in whole genome studies, our approach would be even more powerful when GWAS data is re-analyzed from scratch, limiting the SNPs considered to those in active genomic regions of the cells of interest.

Second, the peak-calling algorithm used (MACS) is not optimal for identifying histone modifications, but was designed for identifying much better-defined transcription factor binding sites. Preliminary analysis showed that we obtained a higher enrichment with other algorithms, such as SICER [48] and ZINBA [49]. Moreover, rather than calling peaks, it may be preferable to identify enhancers based on the profiles of H3K4me1 enrichment together with other chromatin marks as implemented in ChromaSig [50]. The more sophisticated analysis of active genomic regions by the Kellis group, which combined multiple chromatin markers in CD4+ T cells, gave a higher enrichment of disease associated SNPs than our approach of relying solely on H3K4me1 peaks to identify enhancers. We assume that this is both because other *cis*-regulatory elements such as suppressors or isolators may well have similar importance to enhancers, and because even for enhancers, H3K4me1 in combination with other markers may provide a more accurate identification. We expect classifications such as those by the Kellis group to become available for multiple cell types in the near future. Also, the chromatin state classifications could be further tailored to our type of analysis by focusing on those states that show the highest correlation with disease-associated SNPs, and identifying the optimal set of chromatin marks that identifies these regions. Finally, we want to reiterate that asthma-associated SNPs are significantly enriched not only in enhancers and promoters, but also in coding and untranslated regions. The transcription of these regions could further depend on both genetic and epigenetic factors.

In conclusion, we have demonstrated a novel approach to GWAS data analysis that integrates epigenomic information to identify SNPs and cell types contributing to disease. We expect our approach to be broadly applicable, and to further enhance the value of the accumulating information from GWAS of disease. Future work needs to experimentally confirm the functional role of the identified SNPs.

## Materials and Methods

### SNP Datasets

To create a collection of asthma related SNPs, we had all asthma-associated variants (SNPs) downloaded from GWAS Integrator [33] in October 2012. To ensure the completeness of this collection, we then calculated the linked (i.e., in linkage disequilibrium) SNPs for each asthma-associated SNP using HaploReg tool with default parameters [30]. The total number of asthma-associated SNPs in the combined set was 2,510. As a control dataset, we used a common SNPs 135 (uniquely mapped variants that appear in at least 1% of the population) dataset, and subtracted our asthma SNPs collection. Common SNPs 135 were obtained from UCSC Genome Browser [34]. A total number of non-asthma SNPs was determined as 11,327,391. For calculating asthma SNPs enrichment per GWA SNP panel, the SNPs arrays for Affymetrix 6.0, Illumina 550, Illumina 650 and Illumina 1MDuo were downloaded from UCSC Genome Browser [34].

### SNP Enrichment Calculation

To estimate distribution of asthma SNPs in different chromatin states we used chromatin state prediction for CD4+ T-cells by Ernst and Kellis [36]. The predictions were done for hg18 genome assembly. To convert coordinates of our asthma and non-asthma SNPs from hg19 to hg18 assembly we used the lift genome annotations tools from the UCSC genome browser [http://genome.ucsc.edu/cgi-bin/hgLiftOver]. Some non-asthma SNPs where un-mappable by this tool, and discarded from that calculation. For a given chromatin state, we determined the fraction of asthma-associated SNPs located in the corresponding genomic region, and compared it to the fraction of background SNPs to calculate enrichments. Significance was assessed using the chi-square test.

### Identification of H3K4me1 and H3K27Ac Peaks (enhancers)

ChIP-seq H3K4me1 data together with the input data for 37 datasets were obtained from Human Epigenome Atlas, release 5. [http://www.genboree.org/epigenomeatlas/edaccGridViewerPublic.rhtml].

The samples analyzed came from the following cell types. CD4+ T cells: three samples of CD4+ memory T cells, two samples of CD4+ naive T cells, one sample of CD4+ CD25- IL17- T cells, CD4+ CD25- T cells, CD4+ CD25- IL17+ Th17 cells, CD4+ CD25+ CD127- Treg cells; brain: brain germinal cells, brain mid frontal and inferior temporal lobe, two samples of brain hippocampus middle, two samples of brain anterior caudate, two samples of brain substantia nigra and brain cingulate gyrus; three samples of adipose derived mesenchymal (ADM) stem cells; three samples of liver cells; two samples of kidney cells; five samples of adipose nuclei; two samples of breast myoepithelial cells and three samples of skeletal muscle cells. Total number of all analyzed samples is equal to 37; the number of analyzed cell types is equal to 19 because for some cell types data for more than one donor was available. These 19 cell types belonged to eight different tissues.

For each sample we calculated H3K4me1 enriched regions by enrichment of treatment reads under a local background model estimated from control reads by employing the statistical software MACS (v.1.4.1, default parameter settings) [51]. Enriched regions with a p-value ≤1e-5 were defined as enhancers.

All available ChIP-seq H3K27ac data for the samples that contained H3K4me1 datasets were obtained from Human Epigenome Atlas, release 5. Thus, we have analyzed H3K27ac marks for the same CD4+ T cells (9 samples), one sample of brain anterior caudate, one sample of adipose nuclei and one sample of skeletal muscle cells. The twelve cell types mentioned above belong to four different tissues and for each sample we have calculated H3K27ac peaks using exactly the same parameters as we used for H3K4me1 peak calling.

### Comparing H3K4me1 Enhancers between Datasets

In order to compare the enhancers in different datasets we calculated pair-wise Matthews correlation coefficients (MCC), which quantify the overlap of enhancers on a per nucleotide basis. For each pair of samples (datasets) the MCC was calculated using the following formula:
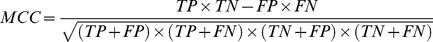
where true positive (TP) was a number of nucleotides present in common enhancers, false positive (FP) and false negative (FN) number of nucleotides in enhancers present in one dataset but not in the other and vice versa, and, finally true negative (TN) was the number of nucleotides in the genome that were not called as enhancers in either dataset. To calculate overlapping and intersecting genome coordinates, we utilized the BEDTools utilities package [52]. The MCC-heatmap was generated by Cluster 3.0 [53] and visualized by TreeView [54].

### Transcription Factor Binding Sites Dataset

To calculate distribution of asthma SNPs within and without Transcription Factor Binding Sites (TFBSs) we have used TFBSs by ChIP-seq data from the ENCODE (Release 2) [55]. The TFBSs ChIP-seq data were obtained from UCSC Genome Browser [34].

## Supporting Information

Figure S1 in File S1 **Asthma-associated SNPs and H3K4me1 (enhancer) enriched regions in the human IKZF3 locus of different cell/tissue types.** From top to bottom, using the UCSC genome browser, are displayed: the gene track (genes), all the SNPs not associated with asthma, the SNPs associated with asthma (red are GWAS-identified SNPs, blue are SNPs in linkage disequilibrium), H3K4me1 ChIP-seq track (green) for different cell/tissue types (named on the left) underlined by the corresponding peak-calling track (black boxes). For the blood CD4+ T cells, peak calling tracks from seven samples/cell-types are displayed. The red box shows an H3K4me1 peak that is present only in CD4+ T cells.
(PDF)
