# Supplementary material for: Predicting Cell Types and Genetic Variations Contributing to Disease by Combining GWAS and Epigenetic Data
Source: PLoS One. 2013 Jan 30;8(1):e54359. doi: 10.1371/journal.pone.0054359 (PMC3559682; doi:10.1371/journal.pone.0054359)
Supplement: Figure S1 in File S1 — Asthma-associated SNPs and H3K4me1 (enhancer) enriched regions in the human IKZF3 locus of different cell/tissue types. From top to bottom, using the UCSC genome browser, are displayed: the gene track (genes), all the SNPs not associated with asthma, the SNPs associated with asthma (red are GWAS-identified SNPs, blue are SNPs in linkage disequilibrium), H3K4me1 ChIP-seq track (green) for different cell/tissue types (named on the left) underlined by the corresponding peak-calling track (black boxes). For the blood CD4+ T cells, peak calling tracks from seven samples/cell-types are displayed. The red box shows an H3K4me1 peak that is present only in CD4+ T cells. (PDF) [file pone.0054359.s001.pdf]

Table S1: Known asthma-associated SNPs from the GWAS integrator database in coordinates of the human genome hg19.

chr1	57126918	57126919	rs2746347
chr1	91288129	91288130	rs3843306
chr1	100335976	100335977	rs17121403
chr1	152492558	152492559	rs4845783
chr1	154426263	154426264	rs4129267
chr1	158932554	158932555	rs1101999
chr1	159174682	159174683	rs2814778
chr1	159175353	159175354	rs12075
chr1	159272059	159272060	rs2251746
chr1	159326879	159326880	rs4656784
chr1	159710516	159710517	rs11265263
chr1	197325907	197325908	rs2786098
chr1	203155881	203155882	rs4950928
chr1	205678125	205678126	rs16856186
chr1	244511175	244511176	rs4658627
chr10	8972017	8972018	rs10508372
chr10	28617634	28617635	rs1148186
chr10	53493472	53493473	rs7922491
chr10	56391302	56391303	rs1937395
chr10	68088507	68088508	rs10762058
chr10	73527046	73527047	rs1867982
chr10	95348181	95348182	rs10882272
chr10	122720973	122720974	rs4752485
chr11	76270682	76270683	rs7130588
chr11	93166730	93166731	rs2658782
chr11	114231254	114231255	rs11214966
chr11	127761665	127761666	rs7927044
chr12	56364320	56364321	rs2069408
chr12	56412486	56412487	rs1701704
chr12	57489708	57489709	rs1059513
chr12	62610859	62610860	rs11615916
chr12	111884607	111884608	rs3184504
chr13	27415672	27415673	rs9319321
chr13	36351765	36351766	rs7328278
chr13	51094113	51094114	rs9316500
chr13	63638328	63638329	rs3119939
chr13	66356340	66356341	rs17077331
chr14	64275811	64275812	rs7144584
chr14	85654018	85654019	rs12436689
chr15	51969667	51969668	rs17525472
chr15	61069987	61069988	rs11071559
chr15	61164198	61164199	rs1902618
chr15	67446784	67446785	rs744910
chr16	27374399	27374400	rs1801275
chr17	13559079	13559080	rs10521233

chr17	38062195	38062196	rs2305480
chr17	38064404	38064405	rs11078927
chr17	38069948	38069949	rs7216389
chr17	38089343	38089344	rs4794820
chr17	38095173	38095174	rs6503525
chr17	38121992	38121993	rs3894194
chr17	38128647	38128648	rs3859192
chr17	70363394	70363395	rs12941150
chr18	29187278	29187279	rs1667255
chr19	53682041	53682042	rs16984547
chr2	4318975	4318976	rs11686135
chr2	8434184	8434185	rs3102947
chr2	42276920	42276921	rs4952590
chr2	46332168	46332169	rs12712969
chr2	81856525	81856526	rs12615721
chr2	102953616	102953617	rs3771180
chr2	102957715	102957716	rs1420101
chr2	102971199	102971200	rs9807989
chr2	102986221	102986222	rs3771166
chr2	201150039	201150040	rs295137
chr2	207840331	207840332	rs4675644
chr2	213824044	213824045	rs12619285
chr2	234814058	234814059	rs10187654
chr20	3827308	3827309	rs4815617
chr20	19363978	19363979	rs2424234
chr20	56663273	56663274	rs6070346
chr21	44156768	44156769	rs9979235
chr21	48019867	48019868	rs881827
chr22	37534033	37534034	rs2284033
chr3	3614886	3614887	rs9815663
chr3	128260549	128260550	rs4857855
chr3	188442479	188442480	rs9290877
chr4	89421085	89421086	rs10516809
chr4	144003158	144003159	rs7686660
chr4	144357736	144357737	rs3805236
chr5	11111770	11111771	rs6884431
chr5	59369793	59369794	rs1588265
chr5	96101943	96101944	rs27524
chr5	110401871	110401872	rs1837253
chr5	110435489	110435490	rs2416257
chr5	131723287	131723288	rs2073643
chr5	131796921	131796922	rs11745587
chr5	131862976	131862977	rs4143832
chr5	131901224	131901225	rs2244012
chr5	131973176	131973177	rs2040704
chr5	131995842	131995843	rs1295686
chr5	131995963	131995964	rs20541

chr5	140700488	140700489	rs10875595
chr5	141445979	141445980	rs6867913
chr6	29849618	29849619	rs115866039
chr6	29923837	29923838	rs116122394
chr6	31446795	31446796	rs116169603
chr6	32076498	32076499	rs115905621
chr6	32155580	32155581	rs114254831
chr6	32184344	32184345	rs115718626
chr6	32338694	32338695	rs116171877
chr6	32358512	32358513	rs114087888
chr6	32414272	32414273	rs114874012
chr6	32433166	32433167	rs115973608
chr6	32625868	32625869	rs9273349
chr6	32658078	32658079	rs114623601
chr6	32681276	32681277	rs114057339
chr6	32687972	32687973	rs114712179
chr6	32961360	32961361	rs114105355
chr6	33042879	33042880	rs115505532
chr6	166534741	166534742	rs6456042
chr7	54181931	54181932	rs6593122
chr7	93538294	93538295	rs180273
chr7	132189688	132189689	rs10808265
chr8	3594905	3594906	rs2623702
chr8	15532584	15532585	rs4831760
chr8	34524002	34524003	rs6987004
chr8	41615137	41615138	rs7006290
chr8	61876043	61876044	rs10104895
chr8	73236809	73236810	rs7006742
chr8	98166912	98166913	rs1835740
chr8	118025644	118025645	rs3019885
chr9	565916	565917	rs7027930
chr9	6190075	6190076	rs1342326
chr9	6193454	6193455	rs2381416
chr9	17487944	17487945	rs2383024
chr9	20098710	20098711	rs16937883
chr9	32433525	32433526	rs10970976
chr9	82039361	82039362	rs2378383
chr9	113300834	113300835	rs1889321
chr9	122258576	122258577	rs10984561

Table S2: All asthma-associate SNPs in hg19 (Known asthma-associated SNPs from the GWAS-integrator database together with their linked variations based on the HaploReg)

chr1	100314837	100314838	rs3753486
chr1	100335976	100335977	rs17121403
chr1	100398599	100398600	rs79427272
chr1	100420558	100420559	rs12128514
chr1	100436132	100436133	rs11166383
chr1	100441443	100441444	rs12141518
chr1	100510454	100510455	rs114364523
chr1	100560263	100560264	rs114469102
chr1	100650208	100650209	rs12120438
chr1	101001770	101001771	rs116815706
chr1	101001772	101001773	rs114478109
chr1	101052428	101052429	rs115937853
chr1	152454590	152454591	rs908922
chr1	152455601	152455602	rs1199150
chr1	152455611	152455612	1:150722236
chr1	152456357	152456358	rs4240882
chr1	152457963	152457964	rs11205010
chr1	152459365	152459366	rs11284452
chr1	152460671	152460672	rs574749
chr1	152474865	152474866	rs2181173
chr1	152475232	152475233	rs6669608
chr1	152475898	152475899	rs7535188
chr1	152476742	152476743	rs499913
chr1	152479175	152479176	rs4845779
chr1	152480653	152480654	rs1415432
chr1	152492558	152492559	rs4845783
chr1	152493153	152493154	rs499697
chr1	152496011	152496012	rs1538084
chr1	152528074	152528075	rs4596895
chr1	152529621	152529622	rs6587688
chr1	152532741	152532742	rs10888499
chr1	154395838	154395839	rs6684439
chr1	154407418	154407419	rs7518199
chr1	154411418	154411419	rs4845622
chr1	154414036	154414037	rs4393147
chr1	154414085	154414086	rs4453032
chr1	154414295	154414296	rs6664201
chr1	154415395	154415396	rs4845372
chr1	154415776	154415777	rs4845623
chr1	154416968	154416969	rs12730036
chr1	154417828	154417829	rs4845373
chr1	154418414	154418415	rs11265613
chr1	154418748	154418749	rs4576655
chr1	154418878	154418879	rs4537545
chr1	154420086	154420087	rs61812598

chr1	154420777	154420778	rs7529229
chr1	154426263	154426264	rs4129267
chr1	154426969	154426970	rs2228145
chr1	158932554	158932555	rs1101999
chr1	159174682	159174683	rs2814778
chr1	159175353	159175354	rs12075
chr1	159258544	159258545	rs2427837
chr1	159272059	159272060	rs2251746
chr1	159287342	159287343	rs36080575
chr1	159318725	159318726	1:157585350
chr1	159326879	159326880	rs4656784
chr1	159372814	159372815	rs34448772
chr1	159410974	159410975	rs4656236
chr1	159411491	159411492	rs61821493
chr1	159430326	159430327	rs7540542
chr1	159710516	159710517	rs11265263
chr1	197312161	197312162	rs2821125
chr1	197315401	197315402	rs2786103
chr1	197316238	197316239	rs1337168
chr1	197316434	197316435	rs1337167
chr1	197320785	197320786	rs2786101
chr1	197322519	197322520	rs2786100
chr1	197325907	197325908	rs2786098
chr1	197328593	197328594	rs2821117
chr1	197329040	197329041	rs2821116
chr1	197329534	197329535	rs2786119
chr1	197330184	197330185	rs2821115
chr1	197332156	197332157	rs2786117
chr1	197333778	197333779	rs2786116
chr1	197337829	197337830	rs10801603
chr1	197339478	197339479	rs6664399
chr1	197343949	197343950	rs2821107
chr1	197351038	197351039	rs2476023
chr1	197351553	197351554	rs2821104
chr1	197352968	197352969	rs2759656
chr1	197363307	197363308	rs2759661
chr1	197365148	197365149	rs2494269
chr1	197366142	197366143	rs2476019
chr1	197368230	197368231	rs2821101
chr1	197368839	197368840	rs2759659
chr1	197379841	197379842	rs2821132
chr1	197392066	197392067	rs12134409
chr1	197396107	197396108	rs17554990
chr1	197408659	197408660	rs6685222
chr1	203155881	203155882	rs4950928
chr1	203158228	203158229	rs946262
chr1	203158971	203158972	rs10920579

chr1	203160125	203160126	rs4950929
chr1	203163366	203163367	rs7541061
chr1	203164847	203164848	rs4950882
chr1	203167870	203167871	rs2486065
chr1	203168091	203168092	rs2494277
chr1	203168473	203168474	rs2153101
chr1	205641342	205641343	rs55915134
chr1	205659490	205659491	rs7520276
chr1	205661366	205661367	rs16856160
chr1	205663212	205663213	rs55858232
chr1	205663574	205663575	rs56973466
chr1	205663955	205663956	rs10900521
chr1	205678125	205678126	rs16856186
chr1	205683590	205683591	1:203950214
chr1	205683625	205683626	1:203950249
chr1	205694258	205694259	rs76104815
chr1	205765039	205765040	rs73080391
chr1	244511175	244511176	rs4658627
chr1	57113000	57113001	rs4912407
chr1	57116501	57116502	rs12073253
chr1	57117969	57117970	rs10889004
chr1	57120671	57120672	rs2746355
chr1	57124190	57124191	rs2746352
chr1	57126874	57126875	rs2796529
chr1	57126918	57126919	rs2746347
chr1	57137885	57137886	rs72666493
chr1	57148620	57148621	rs79906664
chr1	91243375	91243376	rs3843305
chr1	91288129	91288130	rs3843306
chr10	122720973	122720974	rs4752485
chr10	28607595	28607596	rs813081
chr10	28612396	28612397	rs915209
chr10	28612847	28612848	rs1249348
chr10	28612859	28612860	rs1249347
chr10	28613511	28613512	rs1273097
chr10	28614383	28614384	rs2447629
chr10	28615572	28615573	rs1148178
chr10	28615607	28615608	rs1148179
chr10	28615755	28615756	rs1148180
chr10	28616050	28616051	rs1148181
chr10	28616310	28616311	rs34142647
chr10	28616418	28616419	rs1148182
chr10	28616786	28616787	rs34841307
chr10	28617227	28617228	rs1148183
chr10	28617418	28617419	rs1148185
chr10	28617634	28617635	rs1148186
chr10	28619333	28619334	rs1148189

chr10	28619409	28619410	rs34009412
chr10	28619486	28619487	rs811496
chr10	53493472	53493473	rs7922491
chr10	56391302	56391303	rs1937395
chr10	68088347	68088348	rs10762057
chr10	68088507	68088508	rs10762058
chr10	68088605	68088606	rs10762059
chr10	73526467	73526468	rs2166632
chr10	73526522	73526523	rs2166633
chr10	73527046	73527047	rs1867982
chr10	73527675	73527676	rs3747865
chr10	73533805	73533806	10:73203812
chr10	73541280	73541281	rs9299507
chr10	73563838	73563839	rs7896487
chr10	8940393	8940394	rs12253380
chr10	8940961	8940962	rs7893722
chr10	8945901	8945902	rs56169495
chr10	8958586	8958587	rs11255975
chr10	8969451	8969452	rs11255979
chr10	8972017	8972018	rs10508372
chr10	8976043	8976044	rs10905488
chr10	95348181	95348182	rs10882272
chr10	95348904	95348905	rs10882273
chr10	95359864	95359865	rs11187547
chr10	95360026	95360027	rs36014035
chr11	114231254	114231255	rs11214966
chr11	127761665	127761666	rs7927044
chr11	76270682	76270683	rs7130588
chr11	76271004	76271005	rs6592645
chr11	76281509	76281510	rs34350457
chr11	76301254	76301255	rs7926914
chr11	76301315	76301316	rs7927894
chr11	76301374	76301375	rs7927997
chr11	93103457	93103458	rs35803070
chr11	93111477	93111478	rs117384439
chr11	93118839	93118840	rs72972357
chr11	93122127	93122128	rs72972364
chr11	93128119	93128120	11:92767768
chr11	93148664	93148665	rs2608214
chr11	93148816	93148817	rs2608215
chr11	93148947	93148948	rs2658778
chr11	93149171	93149172	rs2608216
chr11	93149276	93149277	rs2446061
chr11	93163136	93163137	rs2658779
chr11	93163798	93163799	rs2605582
chr11	93164956	93164957	rs2605583
chr11	93166367	93166368	rs2658781

chr11	93166730	93166731	rs2658782
chr11	93168884	93168885	rs2019814
chr11	93171861	93171862	rs2605585
chr11	93198982	93198983	rs2658791
chr11	93218354	93218355	rs11604909
chr11	93222310	93222311	rs7116173
chr11	93229160	93229161	rs11601056
chr11	93229318	93229319	rs2605611
chr11	93229755	93229756	rs10466368
chr12	111884607	111884608	rs3184504
chr12	111904370	111904371	rs4766578
chr12	111907430	111907431	rs35350651
chr12	111910218	111910219	rs10774625
chr12	111932799	111932800	rs7137828
chr12	56364320	56364321	rs2069408
chr12	56369505	56369506	rs773107
chr12	56369910	56369911	rs773108
chr12	56374694	56374695	rs773109
chr12	56375136	56375137	rs773110
chr12	56375739	56375740	rs773111
chr12	56375880	56375881	rs773112
chr12	56381915	56381916	rs61937249
chr12	56384686	56384687	rs705698
chr12	56390363	56390364	rs772920
chr12	56390635	56390636	rs705702
chr12	56396767	56396768	rs1689510
chr12	56403576	56403577	rs772921
chr12	56407071	56407072	rs2017445
chr12	56412486	56412487	rs1701704
chr12	56416927	56416928	rs2456973
chr12	56422638	56422639	rs34813703
chr12	56435411	56435412	rs705704
chr12	56435503	56435504	rs705705
chr12	56444631	56444632	rs34415530
chr12	57489708	57489709	rs1059513
chr12	57493726	57493727	rs3024971
chr12	62592380	62592381	12:60878648
chr12	62592744	62592745	rs73131836
chr12	62592826	62592827	rs116095803
chr12	62593228	62593229	12:60879496
chr12	62594791	62594792	rs113968422
chr12	62597367	62597368	rs11609226
chr12	62597528	62597529	rs11609261
chr12	62610859	62610860	rs11615916
chr12	62611436	62611437	rs73133481
chr12	62615907	62615908	rs73133484
chr12	62615974	62615975	12:60902242

chr12	62628187	62628188	rs73135204
chr12	62628401	62628402	rs11615656
chr12	62628557	62628558	rs73135207
chr12	62631537	62631538	12:60917805
chr12	62634291	62634292	rs7308137
chr13	27403323	27403324	rs715958
chr13	27407676	27407677	rs9553905
chr13	27408721	27408722	rs9512417
chr13	27409915	27409916	rs1887594
chr13	27410210	27410211	rs9553906
chr13	27413268	27413269	rs4771033
chr13	27413598	27413599	rs4771034
chr13	27415672	27415673	rs9319321
chr13	27416639	27416640	rs9512420
chr13	27416730	27416731	rs1927512
chr13	27418143	27418144	rs9553911
chr13	27424790	27424791	rs6491160
chr13	27429672	27429673	rs9512427
chr13	27431374	27431375	rs9579046
chr13	36346961	36346962	rs17052784
chr13	36351765	36351766	rs7328278
chr13	51094113	51094114	rs9316500
chr13	51096094	51096095	rs963740
chr13	51126876	51126877	rs1239709
chr13	51133420	51133421	rs2182784
chr13	63638328	63638329	rs3119939
chr13	66356340	66356341	rs17077331
chr14	64275811	64275812	rs7144584
chr14	85616187	85616188	rs36080287
chr14	85620569	85620570	rs12436331
chr14	85623794	85623795	rs12887809
chr14	85624298	85624299	rs34501538
chr14	85624960	85624961	rs12897852
chr14	85626251	85626252	rs17258596
chr14	85628103	85628104	rs12895341
chr14	85630535	85630536	rs12896863
chr14	85630624	85630625	rs12897478
chr14	85631469	85631470	rs4243673
chr14	85634200	85634201	rs12879255
chr14	85636109	85636110	rs1449430
chr14	85652906	85652907	rs34394823
chr14	85653458	85653459	rs12432747
chr14	85654018	85654019	rs12436689
chr14	85654117	85654118	rs12433611
chr14	85655134	85655135	rs4904237
chr14	85656587	85656588	rs67029848
chr14	85660069	85660070	rs734966

chr14	85661982	85661983	rs10145806
chr14	85662420	85662421	rs10135529
chr14	85663779	85663780	rs12895597
chr14	85670478	85670479	rs12883566
chr15	51968173	51968174	rs79883421
chr15	51969667	51969668	rs17525472
chr15	61039386	61039387	rs7172011
chr15	61040590	61040591	rs11632684
chr15	61042866	61042867	rs1351544
chr15	61043134	61043135	rs1817479
chr15	61043377	61043378	rs8025324
chr15	61047058	61047059	rs28378989
chr15	61047143	61047144	rs16943064
chr15	61047897	61047898	rs11637301
chr15	61047925	61047926	rs9920577
chr15	61047929	61047930	rs9920560
chr15	61048199	61048200	rs9920592
chr15	61048212	61048213	rs9920593
chr15	61048577	61048578	rs1020730
chr15	61049242	61049243	rs16943070
chr15	61049539	61049540	rs7162065
chr15	61053950	61053951	rs922783
chr15	61057232	61057233	rs2279294
chr15	61057356	61057357	rs2279293
chr15	61059260	61059261	rs8025689
chr15	61064697	61064698	rs12905602
chr15	61065552	61065553	rs11633029
chr15	61065606	61065607	rs11637671
chr15	61066515	61066516	rs11639084
chr15	61068346	61068347	rs10519067
chr15	61068703	61068704	rs10519068
chr15	61068953	61068954	rs11071557
chr15	61069176	61069177	rs34753162
chr15	61069200	61069201	rs34986765
chr15	61069420	61069421	rs11071558
chr15	61069987	61069988	rs11071559
chr15	61164198	61164199	rs1902618
chr15	67444746	67444747	rs10152544
chr15	67446784	67446785	rs744910
chr15	67447451	67447452	rs11634793
chr15	67458804	67458805	rs28360855
chr16	27374399	27374400	rs1801275
chr17	13559079	13559080	rs10521233
chr17	13563978	13563979	rs116990579
chr17	13571310	13571311	rs78368252
chr17	13571733	13571734	rs35997841
chr17	13572267	13572268	rs80315763

chr17	13572286	13572287	rs75891162
chr17	13572300	13572301	rs74940642
chr17	13572347	13572348	rs74663037
chr17	13572382	13572383	rs76792876
chr17	13572392	13572393	rs74384381
chr17	13572649	13572650	rs12449742
chr17	13572852	13572853	rs12449826
chr17	13573025	13573026	17:13513751
chr17	13573092	13573093	rs76250121
chr17	13573098	13573099	rs79179188
chr17	13573196	13573197	rs75069967
chr17	13573285	13573286	rs77282936
chr17	13573301	13573302	rs75818603
chr17	13573335	13573336	rs76735017
chr17	13573342	13573343	rs76747346
chr17	13573376	13573377	rs74909171
chr17	13573456	13573457	rs75502935
chr17	13573497	13573498	rs77522183
chr17	13573697	13573698	rs76917449
chr17	13573765	13573766	rs79342138
chr17	13573770	13573771	rs78967636
chr17	13573818	13573819	rs76279757
chr17	13573827	13573828	rs76420032
chr17	13573866	13573867	rs79265937
chr17	13573916	13573917	rs113637957
chr17	13573946	13573947	rs79918438
chr17	13574058	13574059	rs76532593
chr17	13574247	13574248	rs12451000
chr17	13574306	13574307	rs12450984
chr17	13585003	13585004	rs76379726
chr17	13585992	13585993	rs77337784
chr17	13589001	13589002	rs75483073
chr17	13589076	13589077	rs77664830
chr17	13601684	13601685	rs2218343
chr17	13603932	13603933	rs74982790
chr17	37910367	37910368	rs2941522
chr17	37912376	37912377	rs12946510
chr17	37916389	37916390	rs35833706
chr17	37921741	37921742	rs907091
chr17	37922258	37922259	rs907092
chr17	37928058	37928059	rs2952140
chr17	37929815	37929816	rs2313430
chr17	37935898	37935899	rs56915530
chr17	37938046	37938047	rs10445308
chr17	37962986	37962987	rs4795395
chr17	37970148	37970149	rs9909593
chr17	37975213	37975214	17:35228740

chr17	37976468	37976469	rs9303277
chr17	38004928	38004929	rs34233420
chr17	38020057	38020058	rs35564481
chr17	38020418	38020419	rs1453559
chr17	38023744	38023745	rs4795397
chr17	38024625	38024626	rs11557466
chr17	38025207	38025208	rs11078925
chr17	38026285	38026286	rs11650661
chr17	38027399	38027400	rs12709365
chr17	38027582	38027583	rs13380815
chr17	38028633	38028634	rs11557467
chr17	38029119	38029120	rs12936231
chr17	38030204	38030205	rs11870965
chr17	38031673	38031674	rs10852935
chr17	38031713	38031714	rs10852936
chr17	38031801	38031802	rs9891174
chr17	38031864	38031865	rs36095411
chr17	38032187	38032188	rs12939457
chr17	38032199	38032200	17:35285726
chr17	38032459	38032460	rs34189114
chr17	38032679	38032680	rs35736272
chr17	38033276	38033277	rs57151617
chr17	38035115	38035116	rs9907088
chr17	38035369	38035370	rs36038753
chr17	38035623	38035624	rs35569035
chr17	38035647	38035648	rs9910826
chr17	38035765	38035766	17:35289292
chr17	38036585	38036586	rs9904624
chr17	38037996	38037997	17:35291523
chr17	38039560	38039561	rs34766440
chr17	38040118	38040119	rs12232497
chr17	38040362	38040363	rs12232498
chr17	38040533	38040534	rs12941333
chr17	38040762	38040763	rs2872507
chr17	38043342	38043343	rs9901146
chr17	38043648	38043649	rs12936409
chr17	38045724	38045725	rs12103884
chr17	38049101	38049102	rs12950209
chr17	38049232	38049233	rs12950743
chr17	38049588	38049589	rs7359623
chr17	38051347	38051348	rs8067378
chr17	38053206	38053207	rs12453507
chr17	38057188	38057189	rs12949100
chr17	38057196	38057197	rs8069176
chr17	38061438	38061439	rs4795399
chr17	38062195	38062196	rs2305480
chr17	38062216	38062217	rs2305479

chr17	38062943	38062944	rs35196450
chr17	38062975	38062976	rs11078926
chr17	38063380	38063381	rs883770
chr17	38063737	38063738	rs62067034
chr17	38063979	38063980	rs36084703
chr17	38064404	38064405	rs11078927
chr17	38064468	38064469	rs11078928
chr17	38066239	38066240	rs2290400
chr17	38066266	38066267	rs1008723
chr17	38066371	38066372	rs56380902
chr17	38067019	38067020	rs4795400
chr17	38068042	38068043	rs869402
chr17	38068513	38068514	rs1011082
chr17	38069075	38069076	rs921650
chr17	38069363	38069364	rs5820308
chr17	38069808	38069809	rs6503524
chr17	38069948	38069949	rs7216389
chr17	38070070	38070071	rs7216558
chr17	38071085	38071086	17:35324612
chr17	38072172	38072173	rs1031458
chr17	38072246	38072247	rs1031460
chr17	38072401	38072402	rs8065777
chr17	38073836	38073837	17:35327363
chr17	38073839	38073840	rs77749396
chr17	38073967	38073968	rs9303279
chr17	38074030	38074031	rs9303280
chr17	38074045	38074046	rs9303281
chr17	38074517	38074518	rs7219923
chr17	38075015	38075016	rs3902920
chr17	38075425	38075426	rs7224129
chr17	38076197	38076198	rs5820310
chr17	38080864	38080865	rs4065275
chr17	38080911	38080912	rs8076131
chr17	38082806	38082807	rs12603332
chr17	38088416	38088417	rs4795405
chr17	38089316	38089317	17:35342843
chr17	38089343	38089344	rs4794820
chr17	38089716	38089717	rs7221814
chr17	38092712	38092713	rs8079416
chr17	38092929	38092930	rs35123741
chr17	38095173	38095174	rs6503525
chr17	38098780	38098781	rs58375553
chr17	38100133	38100134	rs4795406
chr17	38102640	38102641	rs4065986
chr17	38107626	38107627	rs4795408
chr17	38112189	38112190	rs8081462
chr17	38114597	38114598	rs6503526

chr17	38119756	38119757	rs12451084
chr17	38119830	38119831	rs12451100
chr17	38121556	38121557	rs56327227
chr17	38121992	38121993	rs3894194
chr17	38122199	38122200	rs8069202
chr17	38125345	38125346	rs3894193
chr17	38126910	38126911	rs10531136
chr17	38127111	38127112	rs4239225
chr17	38128647	38128648	rs3859192
chr17	38128713	38128714	rs3859191
chr17	38129995	38129996	rs60137005
chr17	38130138	38130139	rs56326707
chr17	38131186	38131187	rs56030650
chr17	38134299	38134300	rs59132767
chr17	38134888	38134889	rs3907022
chr17	70363394	70363395	rs12941150
chr18	29151350	29151351	rs735329
chr18	29154044	29154045	rs1667237
chr18	29155171	29155172	rs4799579
chr18	29155701	29155702	rs17660933
chr18	29155948	29155949	rs11081702
chr18	29156301	29156302	rs2034404
chr18	29156720	29156721	rs1667239
chr18	29156998	29156999	rs1375445
chr18	29157334	29157335	rs1375446
chr18	29157567	29157568	rs2704058
chr18	29159949	29159950	rs6506932
chr18	29160611	29160612	rs7243588
chr18	29160751	29160752	rs1616887
chr18	29160782	29160783	rs11662311
chr18	29161364	29161365	rs2420813
chr18	29161510	29161511	rs4799581
chr18	29167904	29167905	rs1667244
chr18	29169212	29169213	rs34047090
chr18	29169824	29169825	rs3764479
chr18	29172475	29172476	rs723744
chr18	29173679	29173680	rs1080093
chr18	29173794	29173795	rs1080094
chr18	29176459	29176460	rs3764476
chr18	29176872	29176873	rs7235277
chr18	29176970	29176971	rs3794884
chr18	29182351	29182352	rs1791229
chr18	29183586	29183587	rs4799583
chr18	29183811	29183812	rs1473342
chr18	29185267	29185268	rs1791201
chr18	29186127	29186128	rs1667254
chr18	29187165	29187166	rs1611949

chr18	29187278	29187279	rs1667255
chr18	29187572	29187573	rs1791200
chr18	29187740	29187741	rs1791199
chr18	29187888	29187889	rs1791198
chr18	29188780	29188781	rs1667257
chr18	29189217	29189218	rs1667258
chr18	29189246	29189247	rs11664321
chr18	29189456	29189457	rs1791197
chr19	53682041	53682042	rs16984547
chr19	53684221	53684222	rs17272889
chr2	102914213	102914214	rs11690644
chr2	102915965	102915966	rs11406702
chr2	102917238	102917239	rs13418548
chr2	102924614	102924615	rs72823619
chr2	102925434	102925435	rs72823621
chr2	102926361	102926362	rs12470864
chr2	102928616	102928617	rs72823628
chr2	102931533	102931534	rs72823632
chr2	102931611	102931612	rs72823635
chr2	102931762	102931763	2:102298195
chr2	102931825	102931826	rs13020553
chr2	102932511	102932512	rs950881
chr2	102932561	102932562	rs950880
chr2	102934973	102934974	rs10179458
chr2	102936158	102936159	rs72823641
chr2	102937543	102937544	rs10189154
chr2	102937882	102937883	rs10189526
chr2	102939035	102939036	rs13001325
chr2	102939799	102939800	2:102306232
chr2	102949160	102949161	rs12479210
chr2	102950821	102950822	rs13019081
chr2	102953616	102953617	rs3771180
chr2	102954212	102954213	rs72823646
chr2	102954652	102954653	rs13431828
chr2	102955055	102955056	rs13408569
chr2	102955081	102955082	rs13408661
chr2	102957347	102957348	rs10173081
chr2	102957715	102957716	rs1420101
chr2	102960209	102960210	rs3771175
chr2	102966548	102966549	rs10197862
chr2	102967235	102967236	rs13424006
chr2	102967412	102967413	rs6751967
chr2	102967429	102967430	rs6751977
chr2	102967430	102967431	rs6704565
chr2	102967586	102967587	rs6749114
chr2	102967856	102967857	rs6734742
chr2	102967857	102967858	rs6752482

chr2	102967927	102967928	rs4988955
chr2	102968006	102968007	rs4988956
chr2	102968074	102968075	rs4988957
chr2	102968210	102968211	rs10192036
chr2	102968211	102968212	rs10204137
chr2	102968284	102968285	rs4988958
chr2	102968355	102968356	rs10192157
chr2	102968361	102968362	rs10206753
chr2	102968701	102968702	2:102335134
chr2	102968715	102968716	rs72823661
chr2	102968737	102968738	rs72823663
chr2	102968750	102968751	rs67088699
chr2	102968825	102968826	rs5010059
chr2	102969646	102969647	rs13431673
chr2	102969912	102969913	rs11688559
chr2	102969948	102969949	rs11688568
chr2	102969970	102969971	rs11688573
chr2	102969983	102969984	rs11689730
chr2	102970104	102970105	rs11695627
chr2	102970242	102970243	rs7558339
chr2	102970288	102970289	rs7608638
chr2	102970531	102970532	rs34951139
chr2	102970707	102970708	rs34169641
chr2	102970749	102970750	rs4485584
chr2	102970758	102970759	rs4485585
chr2	102971199	102971200	rs9807989
chr2	102971305	102971306	rs9808453
chr2	102971480	102971481	rs4851565
chr2	102971663	102971664	rs9807962
chr2	102972798	102972799	rs4851566
chr2	102974327	102974328	rs56179005
chr2	102974370	102974371	rs7603730
chr2	102974763	102974764	rs10170583
chr2	102975049	102975050	rs10173193
chr2	102976171	102976172	rs10176664
chr2	102977195	102977196	rs6543123
chr2	102977510	102977511	rs11123925
chr2	102977729	102977730	rs10204837
chr2	102978458	102978459	rs3755276
chr2	102979623	102979624	rs9308857
chr2	102980542	102980543	rs1420099
chr2	102981305	102981306	rs10182710
chr2	102982093	102982094	rs13392100
chr2	102984623	102984624	rs1362348
chr2	102986221	102986222	rs3771166
chr2	102986374	102986375	rs1974675
chr2	102987458	102987459	rs6543124

chr2	102990647	102990648	rs17027029
chr2	102997050	102997051	rs72823677
chr2	201110222	201110223	rs295134
chr2	201117943	201117944	rs295119
chr2	201131123	201131124	rs842830
chr2	201131227	201131228	rs4233996
chr2	201136142	201136143	rs4673855
chr2	201139960	201139961	rs4145969
chr2	201141001	201141002	rs295136
chr2	201150039	201150040	rs295137
chr2	201158410	201158411	rs1436164
chr2	201159987	201159988	rs295139
chr2	201163555	201163556	rs295141
chr2	201175243	201175244	rs295149
chr2	201176943	201176944	rs296818
chr2	201184347	201184348	rs34693942
chr2	201184712	201184713	rs159321
chr2	201187774	201187775	rs159320
chr2	201190649	201190650	rs5837730
chr2	201194278	201194279	rs3739119
chr2	201195402	201195403	rs3769471
chr2	201195601	201195602	rs295114
chr2	207840331	207840332	rs4675644
chr2	213817218	213817219	rs2170572
chr2	213818577	213818578	rs7560454
chr2	213824044	213824045	rs12619285
chr2	213826150	213826151	rs10189498
chr2	213827185	213827186	rs6723724
chr2	213830186	213830187	rs6750754
chr2	213830830	213830831	rs55816694
chr2	234814058	234814059	rs10187654
chr2	234815004	234815005	rs4663983
chr2	234820577	234820578	rs6724624
chr2	234821444	234821445	rs11892538
chr2	234821529	234821530	rs11563063
chr2	234823057	234823058	rs6738979
chr2	234824730	234824731	rs1965629
chr2	234825092	234825093	rs10166942
chr2	234825368	234825369	rs2362290
chr2	234825883	234825884	rs1003540
chr2	234826468	234826469	rs11318897
chr2	234826647	234826648	rs10170399
chr2	42217917	42217918	rs1079386
chr2	42218333	42218334	rs1078025
chr2	42219598	42219599	rs12478300
chr2	42225088	42225089	rs4952562
chr2	42227951	42227952	rs1550430

chr2	42230457	42230458	rs12472434
chr2	42230710	42230711	rs58379580
chr2	42230815	42230816	rs58541628
chr2	42232493	42232494	rs12470984
chr2	42232511	42232512	rs12474678
chr2	42233857	42233858	rs75186502
chr2	42235294	42235295	rs74261249
chr2	42235709	42235710	rs79431280
chr2	42236254	42236255	2:42089759
chr2	42236521	42236522	rs4952393
chr2	42238368	42238369	rs12467432
chr2	42243894	42243895	rs4952565
chr2	42247151	42247152	rs75660123
chr2	42249525	42249526	rs9967887
chr2	42252269	42252270	rs76755639
chr2	42252702	42252703	rs971913
chr2	42252875	42252876	rs971914
chr2	42252937	42252938	rs971915
chr2	42253457	42253458	rs4952566
chr2	42254817	42254818	rs4952396
chr2	42258770	42258771	rs72613883
chr2	42259455	42259456	rs12479347
chr2	42259705	42259706	rs12479389
chr2	42259706	42259707	rs61473148
chr2	42260148	42260149	rs12464301
chr2	42260442	42260443	rs12464469
chr2	42260478	42260479	rs12472427
chr2	42260494	42260495	rs12472429
chr2	42261003	42261004	rs4952568
chr2	42261599	42261600	rs6758105
chr2	42261870	42261871	rs12473667
chr2	42262063	42262064	rs10183814
chr2	42262131	42262132	rs10173801
chr2	42263156	42263157	rs11419034
chr2	42263168	42263169	rs4952569
chr2	42263258	42263259	rs4952570
chr2	42263391	42263392	rs4952572
chr2	42263821	42263822	rs4952574
chr2	42264300	42264301	rs13431578
chr2	42264740	42264741	rs4952579
chr2	42264868	42264869	rs72613884
chr2	42265010	42265011	rs72613885
chr2	42265072	42265073	rs13409333
chr2	42265161	42265162	rs13397360
chr2	42265287	42265288	rs13384309
chr2	42265311	42265312	2:42118816
chr2	42265407	42265408	rs13384438

chr2	42265448	42265449	rs13384444
chr2	42265701	42265702	rs13409978
chr2	42265845	42265846	rs4468862
chr2	42266512	42266513	rs4952397
chr2	42266739	42266740	rs4952580
chr2	42267689	42267690	rs72613886
chr2	42267913	42267914	rs72613887
chr2	42267940	42267941	rs72613888
chr2	42267961	42267962	rs72613889
chr2	42268443	42268444	rs4952581
chr2	42268671	42268672	rs4952582
chr2	42268687	42268688	rs4952583
chr2	42268974	42268975	rs12475676
chr2	42269244	42269245	rs12472780
chr2	42269286	42269287	rs4952584
chr2	42269635	42269636	rs4952585
chr2	42269751	42269752	rs4952586
chr2	42270130	42270131	rs4952587
chr2	42270758	42270759	rs79551951
chr2	42271260	42271261	rs12328618
chr2	42271282	42271283	rs114006940
chr2	42271349	42271350	rs10692380
chr2	42271822	42271823	rs4952588
chr2	42272388	42272389	rs735868
chr2	42272661	42272662	rs746580
chr2	42272983	42272984	rs729559
chr2	42273090	42273091	rs729558
chr2	42273549	42273550	rs2077169
chr2	42273828	42273829	rs73930806
chr2	42273839	42273840	rs73930807
chr2	42274062	42274063	rs4075500
chr2	42276350	42276351	rs7583016
chr2	42276404	42276405	rs7569033
chr2	42276451	42276452	rs12470582
chr2	42276605	42276606	rs4952589
chr2	42276920	42276921	rs4952590
chr2	42277178	42277179	rs75514524
chr2	42277208	42277209	rs75884992
chr2	42280065	42280066	rs78572108
chr2	42280969	42280970	rs1550431
chr2	42284109	42284110	rs2289410
chr2	42284883	42284884	rs1044305
chr2	42288319	42288320	rs57299708
chr2	42288768	42288769	rs4952398
chr2	42289121	42289122	rs4952400
chr2	4318975	4318976	rs11686135
chr2	46332168	46332169	rs12712969

chr2	81829436	81829437	rs77703195
chr2	81839995	81839996	rs78342615
chr2	81852121	81852122	rs12470138
chr2	81856525	81856526	rs12615721
chr2	81891496	81891497	rs12468720
chr2	81926380	81926381	rs115642676
chr2	81927820	81927821	rs78953938
chr2	81933179	81933180	rs77253430
chr2	81936494	81936495	rs77611158
chr2	81940864	81940865	rs73939365
chr2	81943715	81943716	rs73939366
chr2	81948510	81948511	rs74769250
chr2	81948610	81948611	rs79620111
chr2	81948801	81948802	rs76839791
chr2	81949115	81949116	rs75201676
chr2	81951297	81951298	rs73939371
chr2	81957165	81957166	rs34426147
chr2	8432131	8432132	rs3097276
chr2	8434184	8434185	rs3102947
chr2	8434947	8434948	rs2931130
chr2	8436978	8436979	rs77941772
chr20	19363978	19363979	rs2424234
chr20	3827308	3827309	rs4815617
chr20	56663273	56663274	rs6070346
chr20	56667124	56667125	rs6092606
chr21	44156768	44156769	rs9979235
chr21	47984562	47984563	rs2839329
chr21	47987166	47987167	rs2839337
chr21	47992400	47992401	rs12483634
chr21	48003192	48003193	rs1047221
chr21	48011047	48011048	rs55863940
chr21	48015321	48015322	rs3804040
chr21	48019867	48019868	rs881827
chr21	48027879	48027880	rs12152107
chr21	48030753	48030754	rs11089082
chr22	37531116	37531117	rs2543537
chr22	37531435	37531436	rs228953
chr22	37532664	37532665	rs228954
chr22	37532698	37532699	rs228955
chr22	37533785	37533786	rs228957
chr22	37533794	37533795	rs228958
chr22	37534033	37534034	rs2284033
chr22	37534946	37534947	rs228960
chr22	37535947	37535948	rs228963
chr22	37536325	37536326	22:35866272
chr22	37536721	37536722	rs63431660
chr22	37537057	37537058	rs228965

chr22	37537513	37537514	rs228966
chr3	128204950	128204951	rs2335052
chr3	128205518	128205519	rs4577488
chr3	128209569	128209570	rs7620408
chr3	128212729	128212730	rs4857898
chr3	128212962	128212963	rs13069288
chr3	128213343	128213344	rs75618320
chr3	128213993	128213994	rs35006112
chr3	128214785	128214786	rs56208556
chr3	128217430	128217431	rs13062436
chr3	128221727	128221728	rs60052547
chr3	128223350	128223351	rs13076142
chr3	128224190	128224191	rs35368203
chr3	128225217	128225218	rs11343621
chr3	128226046	128226047	rs35548916
chr3	128227661	128227662	rs4857900
chr3	128229334	128229335	rs1136444
chr3	128232383	128232384	rs13078209
chr3	128232678	128232679	rs4532151
chr3	128249248	128249249	rs9877627
chr3	128251677	128251678	rs4431128
chr3	128257205	128257206	rs75077124
chr3	128257405	128257406	rs34704109
chr3	128259712	128259713	rs56954786
chr3	128259823	128259824	rs59420157
chr3	128260549	128260550	rs4857855
chr3	128265579	128265580	rs68057450
chr3	188414325	188414326	rs6789732
chr3	188439378	188439379	rs55884274
chr3	188439935	188439936	rs9810196
chr3	188440175	188440176	rs9848114
chr3	188440195	188440196	rs62291267
chr3	188441160	188441161	rs9815874
chr3	188441340	188441341	rs17671338
chr3	188442479	188442480	rs9290877
chr3	188444245	188444246	rs56146114
chr3	188444463	188444464	rs62291291
chr3	188445438	188445439	rs17671434
chr3	188445675	188445676	rs9847345
chr3	188445782	188445783	rs7650683
chr3	188445900	188445901	rs7640368
chr3	188446151	188446152	rs7628937
chr3	188446826	188446827	rs62291294
chr3	188447703	188447704	rs9857993
chr3	188448085	188448086	rs9825149
chr3	188448198	188448199	rs9825301
chr3	188448319	188448320	rs9825482

chr3	3613906	3613907	rs13318567
chr3	3613910	3613911	rs13317823
chr3	3614417	3614418	rs4684396
chr3	3614439	3614440	rs11378499
chr3	3614886	3614887	rs9815663
chr3	3615302	3615303	rs9839458
chr3	3615748	3615749	rs7609670
chr4	143925367	143925368	rs12650233
chr4	143926810	143926811	rs10030917
chr4	143927049	143927050	rs12648157
chr4	143929858	143929859	rs11100764
chr4	143933139	143933140	rs955722
chr4	143938708	143938709	rs11728404
chr4	143939255	143939256	rs12505602
chr4	143939307	143939308	rs28817081
chr4	143948323	143948324	rs1510141
chr4	143949706	143949707	rs58945764
chr4	143955159	143955160	rs11100765
chr4	143955507	143955508	rs10022060
chr4	143956889	143956890	rs10015475
chr4	143957312	143957313	rs33956520
chr4	143960558	143960559	rs2322946
chr4	143960937	143960938	rs3967122
chr4	143963009	143963010	rs12508217
chr4	143965144	143965145	rs9991766
chr4	143968193	143968194	rs13126432
chr4	143968815	143968816	rs4690758
chr4	143971068	143971069	4:144190519
chr4	143971530	143971531	rs4234883
chr4	143972744	143972745	rs4690760
chr4	143972992	143972993	rs13151714
chr4	143975295	143975296	rs13139088
chr4	143976124	143976125	rs11736977
chr4	143976570	143976571	rs4690713
chr4	143977787	143977788	rs28623359
chr4	143978186	143978187	rs4077294
chr4	143978864	143978865	rs4076451
chr4	143979638	143979639	rs66633688
chr4	143981239	143981240	rs4234884
chr4	143982470	143982471	rs4690762
chr4	143982957	143982958	rs10004898
chr4	143983303	143983304	rs10015501
chr4	143986548	143986549	rs10017453
chr4	143987253	143987254	rs11732296
chr4	143997557	143997558	rs4485768
chr4	144000286	144000287	rs4367125
chr4	144002950	144002951	rs7681219

chr4	144003158	144003159	rs7686660
chr4	144003484	144003485	rs28431796
chr4	144003699	144003700	rs57778077
chr4	144003850	144003851	rs28660021
chr4	144003993	144003994	rs28496540
chr4	144004623	144004624	rs59799467
chr4	144004654	144004655	rs57921755
chr4	144005883	144005884	rs10581326
chr4	144008387	144008388	rs4690714
chr4	144008629	144008630	rs4690765
chr4	144008848	144008849	rs66750280
chr4	144009002	144009003	rs12507913
chr4	144009143	144009144	rs4323053
chr4	144010485	144010486	4:144229936
chr4	144010907	144010908	rs4407448
chr4	144011189	144011190	rs28660573
chr4	144011472	144011473	rs28644452
chr4	144011892	144011893	rs58401084
chr4	144012289	144012290	rs35342016
chr4	144014292	144014293	rs72615966
chr4	144021165	144021166	rs11731763
chr4	144357736	144357737	rs3805236
chr4	89421085	89421086	rs10516809
chr5	110401871	110401872	rs1837253
chr5	110402854	110402855	rs10061842
chr5	110403345	110403346	rs4304115
chr5	110404184	110404185	rs17551370
chr5	110408178	110408179	rs10062929
chr5	110412584	110412585	rs11466749
chr5	110412893	110412894	rs11466750
chr5	110413730	110413731	rs11466754
chr5	110416222	110416223	rs7713025
chr5	110418086	110418087	rs55887250
chr5	110427751	110427752	rs72776797
chr5	110428743	110428744	rs7729832
chr5	110428780	110428781	rs7712015
chr5	110429954	110429955	rs1370964
chr5	110431550	110431551	rs56167534
chr5	110433390	110433391	rs13357747
chr5	110433788	110433789	rs59663729
chr5	110434260	110434261	rs7705304
chr5	110435489	110435490	rs2416257
chr5	110435660	110435661	rs1379299
chr5	110441560	110441561	rs2034897
chr5	110442136	110442137	rs10056179
chr5	110444908	110444909	rs10039043
chr5	110458811	110458812	rs13359107

chr5	110464392	110464393	rs11948089
chr5	110467085	110467086	rs6869774
chr5	110467236	110467237	rs6870356
chr5	110467917	110467918	rs7731821
chr5	110469625	110469626	rs11957838
chr5	110471634	110471635	rs7714869
chr5	110472603	110472604	rs13360071
chr5	110473053	110473054	rs11241101
chr5	110476399	110476400	rs439057
chr5	110477701	110477702	rs6898145
chr5	110479177	110479178	rs11958561
chr5	11111770	11111771	rs6884431
chr5	131640535	131640536	rs6860806
chr5	131723287	131723288	rs2073643
chr5	131723288	131723289	rs2073644
chr5	131796921	131796922	rs11745587
chr5	131797546	131797547	rs6894249
chr5	131826852	131826853	rs2549008
chr5	131839617	131839618	rs4705952
chr5	131839859	131839860	5:131867759
chr5	131840081	131840082	rs2706389
chr5	131840395	131840396	rs7736328
chr5	131841308	131841309	rs4546372
chr5	131842174	131842175	rs4705953
chr5	131842220	131842221	rs4705863
chr5	131842279	131842280	rs2706390
chr5	131842326	131842327	rs4705864
chr5	131842564	131842565	rs34596919
chr5	131843188	131843189	rs11434946
chr5	131843305	131843306	rs2706391
chr5	131859510	131859511	rs2706394
chr5	131859704	131859705	rs2548989
chr5	131862030	131862031	rs2548991
chr5	131862976	131862977	rs4143832
chr5	131863243	131863244	rs763595
chr5	131866407	131866408	rs72797338
chr5	131867564	131867565	rs72797340
chr5	131867834	131867835	rs17690122
chr5	131881604	131881605	rs2706401
chr5	131885239	131885240	rs12652920
chr5	131885729	131885730	rs34593653
chr5	131887985	131887986	rs1986009
chr5	131890470	131890471	rs2706334
chr5	131895510	131895511	rs10079653
chr5	131895742	131895743	5:131923642
chr5	131895848	131895849	rs2706338
chr5	131897170	131897171	rs61096033

chr5	131900596	131900597	rs62383710
chr5	131900983	131900984	rs34776903
chr5	131901224	131901225	rs2244012
chr5	131901496	131901497	rs2299015
chr5	131903927	131903928	rs2706345
chr5	131904245	131904246	rs2243677
chr5	131905116	131905117	rs2706347
chr5	131905809	131905810	rs2706348
chr5	131906759	131906760	rs2706349
chr5	131907189	131907190	rs56668723
chr5	131908111	131908112	rs58760622
chr5	131909794	131909795	rs62383714
chr5	131911846	131911847	rs2522414
chr5	131912557	131912558	5:131940457
chr5	131913327	131913328	rs2706353
chr5	131915212	131915213	rs17166050
chr5	131915316	131915317	rs2522403
chr5	131916917	131916918	rs2299013
chr5	131917349	131917350	rs2246176
chr5	131918443	131918444	rs2252775
chr5	131918996	131918997	rs35318926
chr5	131921884	131921885	rs60490216
chr5	131925173	131925174	rs56798121
chr5	131925186	131925187	rs2706362
chr5	131928038	131928039	rs10463893
chr5	131929593	131929594	rs2897443
chr5	131932752	131932753	rs17622991
chr5	131932978	131932979	rs62383750
chr5	131933015	131933016	rs2706370
chr5	131933906	131933907	rs10463894
chr5	131935476	131935477	rs2706372
chr5	131937844	131937845	rs2706374
chr5	131939903	131939904	rs12187537
chr5	131943687	131943688	rs11749376
chr5	131944128	131944129	rs2522394
chr5	131945718	131945719	rs10598599
chr5	131945734	131945735	rs115911400
chr5	131945744	131945745	rs59568225
chr5	131948525	131948526	rs2245460
chr5	131948890	131948891	rs10520114
chr5	131951995	131951996	rs2301713
chr5	131952221	131952222	rs6596086
chr5	131952397	131952398	rs6894017
chr5	131953065	131953066	rs2106984
chr5	131953426	131953427	rs7449456
chr5	131955113	131955114	5:131983013
chr5	131961148	131961149	rs6874184

chr5	131961848	131961849	rs62383757
chr5	131961969	131961970	rs62383758
chr5	131962435	131962436	rs58648981
chr5	131965108	131965109	rs3798135
chr5	131965178	131965179	rs3798134
chr5	131965605	131965606	rs56183820
chr5	131968608	131968609	rs6596087
chr5	131969278	131969279	rs10052993
chr5	131969873	131969874	rs6871536
chr5	131971901	131971902	rs12653750
chr5	131972257	131972258	rs2040703
chr5	131973176	131973177	rs2040704
chr5	131973203	131973204	rs11420290
chr5	131973381	131973382	rs6872131
chr5	131973662	131973663	rs2074369
chr5	131974062	131974063	rs7737470
chr5	131974176	131974177	rs11308531
chr5	131977126	131977127	rs2240032
chr5	131980466	131980467	5:132008366
chr5	131982122	131982123	rs62385261
chr5	131982370	131982371	rs62385262
chr5	131982514	131982515	rs60632435
chr5	131983425	131983426	rs7700346
chr5	131984057	131984058	rs2158177
chr5	131985988	131985989	rs34489085
chr5	131986924	131986925	rs72797378
chr5	131988414	131988415	rs6596090
chr5	131988726	131988727	rs116389633
chr5	131990379	131990380	rs59482112
chr5	131991655	131991656	rs67006560
chr5	131991880	131991881	rs115008099
chr5	131992408	131992409	rs1881457
chr5	131995842	131995843	rs1295686
chr5	131995963	131995964	rs20541
chr5	131996444	131996445	rs1295685
chr5	131996499	131996500	rs848
chr5	131996668	131996669	rs847
chr5	140656397	140656398	rs62378923
chr5	140658362	140658363	rs1363450
chr5	140659105	140659106	rs57396973
chr5	140661931	140661932	rs10062604
chr5	140666493	140666494	rs7703869
chr5	140667302	140667303	rs59933130
chr5	140673428	140673429	rs10067231
chr5	140674733	140674734	rs758346
chr5	140676026	140676027	rs4912747
chr5	140678341	140678342	rs56756237

chr5	140700488	140700489	rs10875595
chr5	140719089	140719090	rs6861047
chr5	141445979	141445980	rs6867913
chr5	141453719	141453720	rs4912799
chr5	141457160	141457161	rs12519447
chr5	59318716	59318717	rs13154041
chr5	59320431	59320432	rs13164245
chr5	59323008	59323009	rs12054868
chr5	59324348	59324349	rs12054971
chr5	59326658	59326659	rs7719879
chr5	59326694	59326695	rs35429428
chr5	59326731	59326732	rs13168606
chr5	59326844	59326845	rs13172650
chr5	59326896	59326897	rs13168887
chr5	59328509	59328510	rs35818757
chr5	59328782	59328783	rs4700354
chr5	59328911	59328912	rs2016324
chr5	59330488	59330489	rs7703245
chr5	59332248	59332249	rs4700355
chr5	59340132	59340133	rs13184009
chr5	59345142	59345143	rs4700357
chr5	59350505	59350506	5:59386263
chr5	59352810	59352811	rs1508864
chr5	59354096	59354097	rs1508859
chr5	59354798	59354799	rs3958941
chr5	59356473	59356474	rs7707407
chr5	59359958	59359959	rs7709983
chr5	59362894	59362895	rs34570495
chr5	59363830	59363831	rs7731007
chr5	59364906	59364907	rs12655292
chr5	59365077	59365078	rs61099545
chr5	59365463	59365464	rs4131157
chr5	59365575	59365576	rs3897743
chr5	59367084	59367085	rs35014288
chr5	59368457	59368458	rs10461667
chr5	59369793	59369794	rs1588265
chr5	59369836	59369837	rs1396475
chr5	59369895	59369896	rs1588266
chr5	59371429	59371430	rs1605730
chr5	59372761	59372762	rs11746704
chr5	59378652	59378653	rs13164971
chr5	59382323	59382324	rs2136203
chr5	59383885	59383886	rs2548659
chr5	59386614	59386615	rs1100918
chr5	59387066	59387067	rs1100917
chr5	59394183	59394184	rs2662444
chr5	59394473	59394474	rs2548658

chr5	59423077	59423078	rs4547891
chr5	59428093	59428094	rs4700366
chr5	59430626	59430627	rs2194257
chr5	59432803	59432804	rs6872268
chr5	59432850	59432851	rs6887486
chr5	59433878	59433879	rs7732524
chr5	59433961	59433962	rs7732670
chr5	59434461	59434462	rs13189802
chr5	59434770	59434771	rs7736514
chr5	59435448	59435449	rs6449456
chr5	59435755	59435756	rs6449457
chr5	59439081	59439082	rs1544791
chr5	59439269	59439270	rs1544790
chr5	59439962	59439963	rs11741663
chr5	59440495	59440496	rs1544788
chr5	59441857	59441858	rs2910840
chr5	59445136	59445137	rs983280
chr5	59449647	59449648	rs2910838
chr5	59452140	59452141	rs2962962
chr5	59453929	59453930	rs2910834
chr5	59456977	59456978	rs2962975
chr5	59461359	59461360	rs1155796
chr5	59461744	59461745	rs2910831
chr5	59464225	59464226	rs2962966
chr5	59484010	59484011	rs2962971
chr5	59484154	59484155	rs2962972
chr5	96101943	96101944	rs27524
chr6	166522623	166522624	rs3127399
chr6	166524416	166524417	rs3099301
chr6	166525451	166525452	rs3127401
chr6	166526498	166526499	rs3099300
chr6	166526586	166526587	rs3099299
chr6	166528912	166528913	rs3099291
chr6	166530657	166530658	rs3099293
chr6	166530658	166530659	rs3127408
chr6	166530991	166530992	rs3099294
chr6	166534144	166534145	rs4709098
chr6	166534388	166534389	rs9459577
chr6	166534442	166534443	rs6924688
chr6	166534489	166534490	rs6920259
chr6	166534635	166534636	rs6456040
chr6	166534657	166534658	rs6940243
chr6	166534678	166534679	rs6456041
chr6	166534741	166534742	rs6456042
chr6	166534775	166534776	rs3127411
chr6	166534825	166534826	rs3099355
chr6	166535060	166535061	rs10716083

chr6	166535560	166535561	rs3127412
chr6	166535727	166535728	rs3127413
chr6	166537087	166537088	rs3127415
chr6	166537411	166537412	rs3127416
chr6	166537733	166537734	rs3127417
chr6	166538175	166538176	rs3127418
chr6	166538526	166538527	rs3127419
chr6	166538547	166538548	rs3127420
chr6	166539058	166539059	rs3127421
chr6	166539069	166539070	rs3127422
chr6	166539087	166539088	rs3127423
chr6	166539089	166539090	rs3127424
chr6	166539226	166539227	rs3127425
chr6	166539538	166539539	rs6927190
chr6	166539593	166539594	rs4343903
chr6	166540844	166540845	rs11413190
chr6	166545909	166545910	rs1900056
chr6	166554169	166554170	rs3099283
chr6	166557338	166557339	rs3099312
chr6	166558949	166558950	rs3127440
chr6	166562035	166562036	rs34771894
chr6	166562853	166562854	rs3127441
chr6	166564997	166564998	rs3054602
chr6	166570765	166570766	rs2277093
chr6	166571156	166571157	rs1134482
chr6	166571163	166571164	rs1134481
chr6	166571442	166571443	rs1056053
chr6	166572667	166572668	rs9348088
chr6	166573446	166573447	rs5881682
chr6	166573711	166573712	rs9295324
chr6	29802044	29802045	rs116338089
chr6	29802689	29802690	rs115108229
chr6	29803525	29803526	rs114282187
chr6	29803879	29803880	rs116568952
chr6	29821566	29821567	rs115055527
chr6	29822138	29822139	rs116664601
chr6	29822260	29822261	rs115364468
chr6	29822412	29822413	rs114522021
chr6	29826091	29826092	rs115328583
chr6	29828659	29828660	rs115137923
chr6	29832723	29832724	rs114052534
chr6	29833793	29833794	rs9280733
chr6	29833839	29833840	rs113809917
chr6	29833947	29833948	rs113380139
chr6	29840145	29840146	rs112349270
chr6	29841071	29841072	rs113365328
chr6	29841279	29841280	rs77637668

chr6	29841482	29841483	rs111593934
chr6	29841504	29841505	rs113446398
chr6	29841989	29841990	rs111943678
chr6	29843200	29843201	rs112278277
chr6	29849618	29849619	rs115866039
chr6	29893153	29893154	rs114428925
chr6	29893234	29893235	rs114796902
chr6	29893559	29893560	rs13214629
chr6	29893620	29893621	6:30001600
chr6	29893625	29893626	rs66531824
chr6	29893664	29893665	rs74295240
chr6	29894204	29894205	rs114217175
chr6	29894225	29894226	rs115615093
chr6	29894797	29894798	rs115783196
chr6	29894990	29894991	rs115261202
chr6	29896044	29896045	rs34608439
chr6	29897258	29897259	rs116702232
chr6	29897349	29897350	rs114321819
chr6	29897693	29897694	rs112918977
chr6	29898225	29898226	rs113312673
chr6	29901782	29901783	rs115236513
chr6	29901897	29901898	rs115252489
chr6	29902560	29902561	6:30010540
chr6	29902898	29902899	rs114611139
chr6	29903012	29903013	rs11312290
chr6	29903235	29903236	rs116565759
chr6	29903566	29903567	rs9259941
chr6	29904071	29904072	rs9280777
chr6	29904407	29904408	rs116631958
chr6	29904491	29904492	rs116651088
chr6	29904981	29904982	rs114380903
chr6	29904994	29904995	6:30012974
chr6	29905223	29905224	rs115231443
chr6	29906379	29906380	rs115753731
chr6	29906723	29906724	rs115354476
chr6	29906980	29906981	rs115032222
chr6	29907122	29907123	rs114540059
chr6	29907123	29907124	rs115484382
chr6	29907158	29907159	rs114964435
chr6	29907163	29907164	rs116331140
chr6	29907200	29907201	rs115892753
chr6	29907312	29907313	rs114199769
chr6	29907450	29907451	rs114582662
chr6	29907834	29907835	rs114260450
chr6	29907949	29907950	rs28993375
chr6	29908092	29908093	rs115592570
chr6	29908261	29908262	rs115409988

chr6	29908425	29908426	rs116458960
chr6	29908468	29908469	rs115314570
chr6	29908679	29908680	6:30016659
chr6	29908803	29908804	rs114682236
chr6	29908966	29908967	rs115354281
chr6	29909043	29909044	rs113376279
chr6	29912226	29912227	6:30020206
chr6	29912462	29912463	rs115842886
chr6	29912594	29912595	rs116303213
chr6	29912795	29912796	rs116397005
chr6	29913134	29913135	rs113282585
chr6	29913733	29913734	rs111727146
chr6	29913816	29913817	rs111867011
chr6	29913880	29913881	rs114145535
chr6	29913911	29913912	rs114069419
chr6	29913956	29913957	rs115359981
chr6	29914010	29914011	rs114733699
chr6	29914056	29914057	rs114388415
chr6	29914088	29914089	rs116243106
chr6	29914303	29914304	rs116240636
chr6	29914398	29914399	rs116786525
chr6	29914827	29914828	rs115470853
chr6	29915123	29915124	rs114690614
chr6	29915300	29915301	rs116543076
chr6	29915407	29915408	rs114089535
chr6	29915765	29915766	rs114024477
chr6	29915766	29915767	rs114749382
chr6	29916252	29916253	rs114095198
chr6	29916580	29916581	rs116151314
chr6	29916649	29916650	rs116566795
chr6	29916654	29916655	rs116279737
chr6	29916678	29916679	rs115445265
chr6	29916680	29916681	rs115980669
chr6	29916685	29916686	rs114889691
chr6	29916764	29916765	rs115942957
chr6	29916792	29916793	rs116461625
chr6	29916960	29916961	rs115987335
chr6	29916961	29916962	rs115493195
chr6	29917032	29917033	rs116451912
chr6	29917037	29917038	rs115850022
chr6	29917052	29917053	rs115953911
chr6	29917167	29917168	rs114412775
chr6	29917353	29917354	rs115384149
chr6	29917438	29917439	rs116708572
chr6	29917495	29917496	rs115737516
chr6	29917502	29917503	rs116835556
chr6	29917799	29917800	rs115264569

chr6	29918172	29918173	rs114905347
chr6	29918287	29918288	rs114325395
chr6	29918309	29918310	rs11421756
chr6	29919962	29919963	rs115263584
chr6	29920399	29920400	rs28993377
chr6	29920674	29920675	rs114010247
chr6	29920950	29920951	rs112010014
chr6	29921033	29921034	rs112183834
chr6	29921699	29921700	rs111469983
chr6	29921862	29921863	rs116382090
chr6	29922666	29922667	rs116617884
chr6	29922739	29922740	rs116675020
chr6	29922852	29922853	rs115042454
chr6	29923420	29923421	rs113425675
chr6	29923837	29923838	rs116122394
chr6	29924219	29924220	rs77843518
chr6	29924298	29924299	rs12205428
chr6	29924358	29924359	rs80040890
chr6	29924388	29924389	rs78231014
chr6	29924888	29924889	rs114845586
chr6	29925126	29925127	rs114959952
chr6	29925971	29925972	rs115661310
chr6	29928486	29928487	rs114298152
chr6	29928837	29928838	rs116200374
chr6	29930495	29930496	rs116282775
chr6	29930705	29930706	rs115405688
chr6	29932737	29932738	rs114452872
chr6	29932864	29932865	rs116772333
chr6	29932896	29932897	rs116666232
chr6	29934696	29934697	rs114012451
chr6	29934906	29934907	rs116330973
chr6	29935575	29935576	rs115278958
chr6	29935589	29935590	rs114902855
chr6	29935842	29935843	rs115186206
chr6	29935848	29935849	rs114120798
chr6	29935890	29935891	rs115640415
chr6	29936306	29936307	rs116251048
chr6	29936350	29936351	rs114603405
chr6	29936407	29936408	rs114461435
chr6	29938454	29938455	rs116121824
chr6	29938486	29938487	rs116212902
chr6	29938506	29938507	rs114867716
chr6	29954363	29954364	rs114738670
chr6	29957068	29957069	rs115216911
chr6	29970926	29970927	rs116837657
chr6	29973525	29973526	rs115092131
chr6	29976788	29976789	rs116774439

chr6	29978351	29978352	rs116641868
chr6	29984251	29984252	rs116693421
chr6	29985629	29985630	rs116554894
chr6	29987183	29987184	rs114336030
chr6	29990937	29990938	rs115197355
chr6	30005753	30005754	rs116373137
chr6	30006764	30006765	rs115678795
chr6	30008745	30008746	rs114740580
chr6	30010079	30010080	rs10580504
chr6	30010491	30010492	rs116676427
chr6	30015593	30015594	rs115677230
chr6	30016675	30016676	rs114960138
chr6	30040083	30040084	rs115819718
chr6	30040290	30040291	rs115550514
chr6	30040602	30040603	rs115441264
chr6	30047402	30047403	rs115144807
chr6	31446795	31446796	rs116169603
chr6	31447134	31447135	rs115912299
chr6	31447587	31447588	rs114285281
chr6	31448624	31448625	rs116793141
chr6	31449268	31449269	rs114032620
chr6	31449993	31449994	rs115094172
chr6	31451679	31451680	rs116275086
chr6	31451848	31451849	rs115789403
chr6	31452715	31452716	rs114437271
chr6	31453112	31453113	rs114654571
chr6	31458040	31458041	rs115690672
chr6	31459482	31459483	rs116662362
chr6	32076498	32076499	rs115905621
chr6	32094592	32094593	rs116089240
chr6	32155580	32155581	rs114254831
chr6	32184344	32184345	rs115718626
chr6	32184573	32184574	rs114708290
chr6	32186192	32186193	rs114677078
chr6	32186244	32186245	rs114221506
chr6	32186871	32186872	rs116616321
chr6	32338282	32338283	rs115983894
chr6	32338694	32338695	rs116171877
chr6	32339896	32339897	rs115597234
chr6	32342821	32342822	rs116454000
chr6	32343713	32343714	rs114752560
chr6	32346771	32346772	rs114845955
chr6	32348308	32348309	rs115825744
chr6	32350106	32350107	rs114210228
chr6	32354643	32354644	rs114520020
chr6	32358285	32358286	rs115112751
chr6	32358512	32358513	rs114087888

chr6	32359762	32359763	rs115295735
chr6	32365579	32365580	rs116797542
chr6	32365839	32365840	rs116628567
chr6	32377715	32377716	6:32485694
chr6	32380397	32380398	rs9279640
chr6	32380716	32380717	rs114693266
chr6	32387220	32387221	rs116657475
chr6	32393061	32393062	rs115872036
chr6	32393160	32393161	rs116279025
chr6	32394250	32394251	rs114893192
chr6	32394910	32394911	rs116385426
chr6	32397812	32397813	rs114193276
chr6	32398524	32398525	rs114653252
chr6	32400343	32400344	rs116330858
chr6	32402704	32402705	rs114618774
chr6	32414272	32414273	rs114874012
chr6	32415079	32415080	rs115365372
chr6	32415425	32415426	rs114056809
chr6	32415787	32415788	rs116264113
chr6	32428061	32428062	rs114975350
chr6	32428078	32428079	rs114455794
chr6	32428114	32428115	rs115918645
chr6	32428185	32428186	rs116231999
chr6	32428284	32428285	rs114865495
chr6	32428714	32428715	rs114800139
chr6	32428803	32428804	rs116106354
chr6	32428861	32428862	rs115948541
chr6	32429086	32429087	rs114078245
chr6	32429244	32429245	rs115748304
chr6	32429326	32429327	rs115120946
chr6	32429476	32429477	rs114357498
chr6	32429642	32429643	rs114240154
chr6	32429671	32429672	rs116531958
chr6	32429674	32429675	rs116225404
chr6	32429718	32429719	rs115532780
chr6	32429757	32429758	rs116814466
chr6	32429803	32429804	rs114858310
chr6	32429821	32429822	rs115977628
chr6	32429824	32429825	rs114706637
chr6	32429893	32429894	rs115710432
chr6	32430166	32430167	rs114324670
chr6	32430325	32430326	rs116154425
chr6	32430361	32430362	rs115371644
chr6	32430507	32430508	rs114664081
chr6	32430603	32430604	rs116580588
chr6	32430728	32430729	rs114678126
chr6	32430751	32430752	rs116556248

chr6	32430799	32430800	rs114126185
chr6	32430813	32430814	rs114393349
chr6	32430866	32430867	rs115433728
chr6	32430974	32430975	rs116115875
chr6	32431104	32431105	rs114634027
chr6	32431123	32431124	rs116813153
chr6	32431128	32431129	rs114854965
chr6	32431291	32431292	rs115052056
chr6	32431305	32431306	rs114274063
chr6	32431357	32431358	rs114184424
chr6	32431622	32431623	rs116153127
chr6	32431639	32431640	rs116737393
chr6	32431704	32431705	rs114332144
chr6	32431827	32431828	rs115026185
chr6	32431832	32431833	rs116506231
chr6	32431885	32431886	rs116554358
chr6	32431895	32431896	rs115951072
chr6	32431913	32431914	rs114349922
chr6	32431921	32431922	rs115660922
chr6	32431953	32431954	rs115242711
chr6	32431961	32431962	rs114002140
chr6	32431964	32431965	rs115848988
chr6	32431978	32431979	rs116650916
chr6	32431979	32431980	rs114048983
chr6	32431994	32431995	rs116408942
chr6	32432006	32432007	rs115709913
chr6	32432043	32432044	rs114264405
chr6	32432044	32432045	rs114968252
chr6	32432048	32432049	rs115777539
chr6	32432076	32432077	rs114354639
chr6	32432180	32432181	rs115179728
chr6	32432232	32432233	rs116191381
chr6	32432233	32432234	rs115253977
chr6	32432370	32432371	rs114963240
chr6	32432379	32432380	rs9281834
chr6	32432381	32432382	rs116251982
chr6	32432405	32432406	rs115428496
chr6	32432519	32432520	rs116832041
chr6	32432555	32432556	rs114756817
chr6	32432581	32432582	rs115332348
chr6	32432597	32432598	rs114008741
chr6	32432614	32432615	rs116115602
chr6	32432645	32432646	rs115406947
chr6	32432663	32432664	rs115239114
chr6	32432688	32432689	rs9281835
chr6	32432834	32432835	rs114260710
chr6	32432857	32432858	rs114910984

chr6	32432991	32432992	rs34335527
chr6	32432992	32432993	rs114417017
chr6	32433166	32433167	rs115973608
chr6	32433191	32433192	rs114701036
chr6	32433653	32433654	rs116196327
chr6	32434239	32434240	rs115367740
chr6	32434348	32434349	rs116301112
chr6	32434360	32434361	rs115234248
chr6	32434638	32434639	rs116663036
chr6	32434668	32434669	rs9281837
chr6	32434715	32434716	rs9281838
chr6	32434849	32434850	rs116003090
chr6	32434873	32434874	rs116352802
chr6	32435106	32435107	rs9279691
chr6	32435375	32435376	rs115293958
chr6	32436216	32436217	rs114624083
chr6	32438541	32438542	rs114100277
chr6	32438597	32438598	rs114024839
chr6	32438647	32438648	rs114326983
chr6	32438676	32438677	rs116234214
chr6	32438849	32438850	rs116807284
chr6	32438866	32438867	rs115035228
chr6	32439028	32439029	rs114474838
chr6	32439047	32439048	rs116544855
chr6	32439067	32439068	rs115311837
chr6	32439076	32439077	rs114440274
chr6	32439095	32439096	rs116375348
chr6	32439107	32439108	rs115462856
chr6	32439322	32439323	rs116230731
chr6	32439392	32439393	rs115425034
chr6	32439507	32439508	rs115119110
chr6	32439752	32439753	rs115383757
chr6	32439827	32439828	rs115446493
chr6	32439864	32439865	rs116813852
chr6	32440676	32440677	rs9281842
chr6	32440770	32440771	rs115771918
chr6	32441007	32441008	rs114951502
chr6	32441099	32441100	rs114475122
chr6	32441391	32441392	rs28986194
chr6	32443640	32443641	rs116469172
chr6	32444454	32444455	rs115793823
chr6	32445599	32445600	rs115974809
chr6	32446970	32446971	rs116175877
chr6	32447057	32447058	rs114723578
chr6	32447119	32447120	rs115550413
chr6	32447153	32447154	rs114718696
chr6	32447784	32447785	rs3039403

chr6	32447815	32447816	rs116142178
chr6	32449973	32449974	rs116332467
chr6	32451677	32451678	rs116635588
chr6	32451901	32451902	rs115786583
chr6	32452280	32452281	rs115969637
chr6	32452505	32452506	rs114926876
chr6	32454060	32454061	rs114715869
chr6	32454115	32454116	rs114714269
chr6	32455170	32455171	rs115244821
chr6	32455259	32455260	rs112852408
chr6	32455438	32455439	rs111388755
chr6	32457347	32457348	6:32565326
chr6	32457773	32457774	6:32565752
chr6	32458279	32458280	rs75781111
chr6	32459108	32459109	rs114375160
chr6	32459274	32459275	rs114193508
chr6	32459416	32459417	rs77945341
chr6	32459844	32459845	6:32567823
chr6	32460013	32460014	rs116775838
chr6	32460272	32460273	6:32568251
chr6	32460406	32460407	rs77655711
chr6	32460617	32460618	6:32568596
chr6	32461217	32461218	6:32569196
chr6	32469887	32469888	6:32577866
chr6	32470357	32470358	rs113291983
chr6	32472038	32472039	6:32580017
chr6	32472080	32472081	rs75989399
chr6	32472194	32472195	rs77749841
chr6	32473627	32473628	rs114313885
chr6	32473635	32473636	rs114666889
chr6	32474960	32474961	rs111832132
chr6	32478533	32478534	rs114899036
chr6	32479243	32479244	rs113008589
chr6	32479870	32479871	6:32587849
chr6	32482055	32482056	rs111251172
chr6	32482409	32482410	6:32590388
chr6	32482455	32482456	6:32590434
chr6	32484548	32484549	rs113437968
chr6	32484905	32484906	6:32592884
chr6	32484923	32484924	rs114586378
chr6	32485523	32485524	rs77867566
chr6	32485588	32485589	rs112528767
chr6	32485704	32485705	rs115308342
chr6	32486817	32486818	rs114561288
chr6	32487825	32487826	rs116822900
chr6	32487839	32487840	rs114291341
chr6	32488859	32488860	rs112968327

chr6	32491230	32491231	rs114158220
chr6	32492780	32492781	rs115904597
chr6	32493164	32493165	rs63563560
chr6	32494174	32494175	rs115712333
chr6	32500847	32500848	6:32608826
chr6	32501612	32501613	rs112992227
chr6	32502321	32502322	rs78182729
chr6	32502870	32502871	rs74955625
chr6	32502884	32502885	rs77572094
chr6	32502933	32502934	rs75211336
chr6	32503335	32503336	rs112863512
chr6	32503374	32503375	rs112259157
chr6	32503397	32503398	rs113126384
chr6	32503466	32503467	rs111864865
chr6	32503560	32503561	rs79293578
chr6	32503566	32503567	rs79220160
chr6	32503657	32503658	rs112177836
chr6	32503696	32503697	rs75730517
chr6	32503920	32503921	rs74323244
chr6	32504174	32504175	rs111929170
chr6	32505470	32505471	rs112796795
chr6	32505642	32505643	6:32613621
chr6	32505761	32505762	rs77931438
chr6	32505975	32505976	6:32613954
chr6	32507210	32507211	rs116802768
chr6	32508371	32508372	6:32616350
chr6	32508411	32508412	6:32616390
chr6	32509238	32509239	6:32617217
chr6	32509293	32509294	rs79207037
chr6	32509454	32509455	rs112490248
chr6	32509550	32509551	rs113686014
chr6	32509934	32509935	rs59350153
chr6	32510133	32510134	rs56942249
chr6	32510581	32510582	rs79005639
chr6	32510601	32510602	rs112895453
chr6	32510650	32510651	rs112467274
chr6	32511008	32511009	rs115591310
chr6	32511649	32511650	rs111797109
chr6	32511752	32511753	6:32619731
chr6	32512048	32512049	rs112582570
chr6	32512309	32512310	rs112251372
chr6	32512574	32512575	6:32620553
chr6	32513284	32513285	rs113542603
chr6	32513487	32513488	6:32621466
chr6	32513623	32513624	6:32621602
chr6	32514161	32514162	rs113484779
chr6	32514499	32514500	rs62405631

chr6	32517302	32517303	rs111403760
chr6	32517649	32517650	rs112651699
chr6	32517667	32517668	rs112659139
chr6	32517726	32517727	rs111838566
chr6	32517988	32517989	rs74636204
chr6	32518103	32518104	rs75515332
chr6	32518168	32518169	6:32626147
chr6	32518519	32518520	6:32626498
chr6	32518665	32518666	6:32626644
chr6	32518703	32518704	rs112458756
chr6	32519284	32519285	rs35916739
chr6	32520271	32520272	rs111513373
chr6	32520459	32520460	rs113521434
chr6	32520537	32520538	6:32628516
chr6	32521619	32521620	6:32629598
chr6	32521840	32521841	rs75513198
chr6	32521924	32521925	6:32629903
chr6	32521983	32521984	rs112296425
chr6	32522121	32522122	6:32630100
chr6	32522185	32522186	rs114773114
chr6	32523395	32523396	rs114022150
chr6	32524950	32524951	rs114899212
chr6	32525043	32525044	6:32633022
chr6	32525067	32525068	rs114074434
chr6	32525218	32525219	rs114742549
chr6	32525222	32525223	rs115187291
chr6	32525228	32525229	rs115546388
chr6	32538841	32538842	6:32646820
chr6	32541519	32541520	rs9269421
chr6	32541598	32541599	rs115929456
chr6	32542125	32542126	rs9281857
chr6	32544414	32544415	rs35100578
chr6	32548655	32548656	6:32656634
chr6	32549192	32549193	rs35247783
chr6	32550370	32550371	6:32658349
chr6	32550549	32550550	rs28724008
chr6	32550613	32550614	rs9269863
chr6	32550904	32550905	rs28724033
chr6	32553133	32553134	6:32661112
chr6	32554724	32554725	rs35984981
chr6	32555376	32555377	rs34977583
chr6	32555557	32555558	rs9270161
chr6	32559780	32559781	rs28366267
chr6	32559933	32559934	rs28366270
chr6	32560287	32560288	rs28366283
chr6	32560431	32560432	rs28366288
chr6	32560510	32560511	rs28359874

chr6	32560714	32560715	rs28366294
chr6	32560715	32560716	rs28366295
chr6	32560726	32560727	rs28366296
chr6	32560828	32560829	rs28366297
chr6	32560858	32560859	rs28366298
chr6	32560869	32560870	rs28366299
chr6	32560874	32560875	rs28366300
chr6	32560882	32560883	rs28366301
chr6	32560933	32560934	rs28366302
chr6	32560962	32560963	rs28366303
chr6	32561047	32561048	rs28366309
chr6	32561127	32561128	rs28366310
chr6	32561136	32561137	rs28366311
chr6	32561160	32561161	rs9270582
chr6	32561174	32561175	rs28366312
chr6	32561185	32561186	rs28366313
chr6	32561200	32561201	rs28366314
chr6	32561206	32561207	rs28366315
chr6	32561245	32561246	rs28366316
chr6	32561410	32561411	rs28366317
chr6	32561494	32561495	rs28366319
chr6	32561608	32561609	rs78493528
chr6	32561658	32561659	rs75676849
chr6	32561677	32561678	rs115987022
chr6	32561765	32561766	rs113916850
chr6	32561818	32561819	rs28366329
chr6	32561845	32561846	rs28366330
chr6	32561879	32561880	rs28366331
chr6	32561910	32561911	rs28366335
chr6	32561955	32561956	rs28366336
chr6	32569690	32569691	rs477515
chr6	32569851	32569852	rs622137
chr6	32570399	32570400	rs2516049
chr6	32570400	32570401	rs2454138
chr6	32570879	32570880	rs556025
chr6	32571115	32571116	rs116213307
chr6	32571725	32571726	rs9281923
chr6	32572029	32572030	rs526784
chr6	32579127	32579128	rs9281940
chr6	32581921	32581922	rs114892052
chr6	32584418	32584419	6:32692397
chr6	32585464	32585465	6:32693443
chr6	32587116	32587117	rs28383313
chr6	32587612	32587613	rs9271408
chr6	32587627	32587628	rs9271409
chr6	32587660	32587661	rs9271411
chr6	32587811	32587812	rs9271416

chr6	32587972	32587973	rs9271426
chr6	32588001	32588002	rs9271430
chr6	32588192	32588193	rs9271438
chr6	32588311	32588312	6:32696290
chr6	32588553	32588554	rs9271465
chr6	32588661	32588662	rs9271470
chr6	32588699	32588700	rs9271472
chr6	32588706	32588707	rs9271474
chr6	32588830	32588831	rs80180464
chr6	32588952	32588953	rs78474935
chr6	32588969	32588970	rs115180982
chr6	32588999	32589000	rs9271488
chr6	32589281	32589282	rs113353196
chr6	32589325	32589326	rs113060503
chr6	32589482	32589483	rs111375871
chr6	32589667	32589668	rs9271516
chr6	32589720	32589721	6:32697699
chr6	32589770	32589771	rs9271520
chr6	32589782	32589783	rs9271521
chr6	32590028	32590029	rs9271540
chr6	32590086	32590087	rs9279819
chr6	32590090	32590091	rs9271542
chr6	32590119	32590120	rs9271544
chr6	32590247	32590248	rs9271549
chr6	32590380	32590381	rs77599748
chr6	32590392	32590393	rs74638570
chr6	32590482	32590483	rs111554896
chr6	32590589	32590590	rs113150735
chr6	32590611	32590612	rs113104509
chr6	32590773	32590774	rs113888335
chr6	32590779	32590780	rs112372067
chr6	32590783	32590784	rs5875381
chr6	32590869	32590870	rs116196531
chr6	32590878	32590879	rs114700763
chr6	32591127	32591128	rs114754567
chr6	32591172	32591173	rs114158560
chr6	32591429	32591430	rs113518441
chr6	32591447	32591448	rs115472351
chr6	32591565	32591566	rs112574272
chr6	32591596	32591597	rs112175921
chr6	32591920	32591921	rs9271624
chr6	32591970	32591971	rs9271628
chr6	32592123	32592124	rs9271637
chr6	32592228	32592229	rs9271643
chr6	32592336	32592337	rs9271644
chr6	32592601	32592602	rs9271660
chr6	32592604	32592605	rs9271661

chr6	32592844	32592845	rs9271674
chr6	32592897	32592898	rs9271677
chr6	32592922	32592923	rs9271682
chr6	32592969	32592970	rs9271685
chr6	32593032	32593033	rs74827550
chr6	32593166	32593167	rs4587163
chr6	32593464	32593465	rs9271718
chr6	32593521	32593522	rs9271722
chr6	32593638	32593639	rs9271734
chr6	32593648	32593649	rs9271735
chr6	32593687	32593688	rs9271736
chr6	32594021	32594022	rs9271761
chr6	32594027	32594028	rs9271762
chr6	32594158	32594159	rs9271764
chr6	32594165	32594166	rs9271765
chr6	32594175	32594176	rs9271767
chr6	32594187	32594188	rs9271768
chr6	32594216	32594217	rs9271769
chr6	32594303	32594304	rs9271773
chr6	32594340	32594341	rs9271776
chr6	32594402	32594403	rs113138877
chr6	32594440	32594441	rs113414682
chr6	32594659	32594660	6:32702638
chr6	32595096	32595097	rs112526259
chr6	32596474	32596475	rs114515013
chr6	32596837	32596838	rs35828368
chr6	32603865	32603866	rs76650283
chr6	32604007	32604008	rs9281995
chr6	32604331	32604332	rs9279877
chr6	32604393	32604394	rs9272347
chr6	32604869	32604870	rs9281999
chr6	32605100	32605101	rs9272420
chr6	32605294	32605295	rs9272433
chr6	32605645	32605646	rs9272463
chr6	32605971	32605972	rs9272485
chr6	32605981	32605982	rs9282003
chr6	32606051	32606052	rs9272492
chr6	32606941	32606942	rs28383365
chr6	32607252	32607253	rs28383380
chr6	32607319	32607320	rs28383382
chr6	32607367	32607368	rs28383383
chr6	32607440	32607441	rs28359903
chr6	32607897	32607898	rs28359904
chr6	32607944	32607945	rs9272619
chr6	32607949	32607950	rs28383400
chr6	32607972	32607973	rs28383401
chr6	32607984	32607985	rs28383402

chr6	32608034	32608035	rs28383403
chr6	32608391	32608392	rs28383903
chr6	32608416	32608417	rs28383413
chr6	32608536	32608537	rs28383418
chr6	32609055	32609056	rs28383441
chr6	32611180	32611181	rs1064993
chr6	32611356	32611357	rs9272980
chr6	32611536	32611537	rs9273007
chr6	32611649	32611650	rs9273014
chr6	32611768	32611769	rs9273026
chr6	32611897	32611898	rs9273036
chr6	32612160	32612161	rs9273062
chr6	32612165	32612166	rs9273063
chr6	32612451	32612452	6:32720430
chr6	32612575	32612576	rs9273103
chr6	32620159	32620160	rs17843747
chr6	32621900	32621901	rs17613299
chr6	32621907	32621908	rs17613305
chr6	32622837	32622838	rs9273302
chr6	32623101	32623102	rs9273322
chr6	32623156	32623157	6:32731135
chr6	32625868	32625869	rs9273349
chr6	32626345	32626346	rs115198367
chr6	32642468	32642469	rs116170168
chr6	32649179	32649180	rs116620935
chr6	32649346	32649347	rs116724396
chr6	32650255	32650256	rs115160772
chr6	32650549	32650550	rs116482027
chr6	32651403	32651404	rs114627629
chr6	32651867	32651868	rs116129881
chr6	32652468	32652469	rs115715553
chr6	32653731	32653732	rs116756126
chr6	32654256	32654257	rs115224792
chr6	32655404	32655405	rs114897141
chr6	32657081	32657082	rs114323889
chr6	32657254	32657255	rs115383573
chr6	32657280	32657281	rs115144330
chr6	32657451	32657452	rs115206228
chr6	32657816	32657817	rs114249316
chr6	32658078	32658079	rs114623601
chr6	32658350	32658351	rs115643108
chr6	32658365	32658366	rs116630088
chr6	32658374	32658375	rs114158461
chr6	32658512	32658513	rs116737275
chr6	32658714	32658715	rs114901942
chr6	32658726	32658727	rs114059691
chr6	32658732	32658733	rs115142413

chr6	32658934	32658935	rs116321448
chr6	32659821	32659822	rs115144232
chr6	32660365	32660366	rs115982341
chr6	32660706	32660707	rs114312235
chr6	32660900	32660901	6:32768879
chr6	32661514	32661515	rs115478468
chr6	32661640	32661641	rs116084993
chr6	32662723	32662724	rs115204828
chr6	32663359	32663360	rs114224180
chr6	32663577	32663578	rs116321787
chr6	32663771	32663772	rs115204718
chr6	32663918	32663919	rs115988487
chr6	32664092	32664093	rs114763381
chr6	32664278	32664279	rs115840989
chr6	32664676	32664677	rs115162422
chr6	32664759	32664760	rs114467938
chr6	32665596	32665597	rs116663678
chr6	32665812	32665813	rs114786695
chr6	32681276	32681277	rs114057339
chr6	32687972	32687973	rs114712179
chr6	32700760	32700761	rs115655175
chr6	32709963	32709964	rs115434195
chr6	32709989	32709990	rs116593970
chr6	32710009	32710010	rs116457257
chr6	32957388	32957389	rs116132491
chr6	32959719	32959720	6:33067698
chr6	32961360	32961361	rs114105355
chr6	32962881	32962882	rs115569272
chr6	33024813	33024814	rs115673219
chr6	33024822	33024823	rs115407957
chr6	33024907	33024908	rs114935065
chr6	33024971	33024972	rs116486262
chr6	33025043	33025044	rs114930606
chr6	33025049	33025050	rs114142782
chr6	33025385	33025386	rs116176189
chr6	33025711	33025712	rs116400397
chr6	33025739	33025740	rs115820704
chr6	33026184	33026185	rs114861986
chr6	33026387	33026388	rs114747995
chr6	33026432	33026433	rs115254361
chr6	33028727	33028728	rs114989950
chr6	33029159	33029160	rs115882270
chr6	33031416	33031417	rs114626190
chr6	33031538	33031539	rs115898971
chr6	33031611	33031612	rs114129321
chr6	33031688	33031689	rs115795025
chr6	33031916	33031917	rs115731617

chr6	33032622	33032623	rs115092330
chr6	33032852	33032853	rs115713637
chr6	33033312	33033313	rs114522401
chr6	33033638	33033639	rs114866421
chr6	33034472	33034473	rs114657860
chr6	33037293	33037294	rs115768631
chr6	33037954	33037955	rs115478098
chr6	33037956	33037957	rs114508533
chr6	33038032	33038033	rs116370068
chr6	33038116	33038117	rs116470580
chr6	33038133	33038134	rs114757491
chr6	33038801	33038802	rs116140561
chr6	33038981	33038982	rs116686655
chr6	33039195	33039196	rs116041988
chr6	33039651	33039652	rs115380031
chr6	33039680	33039681	rs114565863
chr6	33039693	33039694	rs115056735
chr6	33039696	33039697	rs116167529
chr6	33039898	33039899	rs116574766
chr6	33040043	33040044	rs115416482
chr6	33040477	33040478	rs114308029
chr6	33040653	33040654	rs116711188
chr6	33042367	33042368	rs114917710
chr6	33042405	33042406	rs116254741
chr6	33042491	33042492	rs115189254
chr6	33042879	33042880	rs115505532
chr6	33044187	33044188	rs115475655
chr6	33044257	33044258	rs116543263
chr6	33044387	33044388	rs114118792
chr7	132185853	132185854	rs77902573
chr7	132187402	132187403	rs3847115
chr7	132189688	132189689	rs10808265
chr7	132190234	132190235	rs34244529
chr7	132195217	132195218	rs35813265
chr7	132196752	132196753	rs4728258
chr7	132218940	132218941	rs11769821
chr7	132219323	132219324	rs56031339
chr7	132226504	132226505	rs34936676
chr7	54154000	54154001	7:54121495
chr7	54160751	54160752	rs6946668
chr7	54163221	54163222	rs6944870
chr7	54169840	54169841	rs7459150
chr7	54174127	54174128	rs17560456
chr7	54181560	54181561	rs10707655
chr7	54181931	54181932	rs6593122
chr7	54183654	54183655	rs73122354
chr7	54184060	54184061	rs2177549

chr7	54186166	54186167	rs1916972
chr7	54187364	54187365	rs68178910
chr7	54187498	54187499	rs66529760
chr7	54187664	54187665	rs67719253
chr7	54188503	54188504	rs73124131
chr7	54189502	54189503	rs5009546
chr7	54189518	54189519	rs5009545
chr7	54189521	54189522	rs5009544
chr7	54191188	54191189	rs10230619
chr7	54193185	54193186	rs4466358
chr7	54193196	54193197	rs4543496
chr7	54194120	54194121	rs7797113
chr7	54194726	54194727	rs6968968
chr7	54197084	54197085	7:54164579
chr7	54199430	54199431	rs77979891
chr7	54202304	54202305	rs6960827
chr7	54202396	54202397	rs6979442
chr7	54206714	54206715	rs2049411
chr7	93531045	93531046	rs180282
chr7	93533838	93533839	rs180278
chr7	93535059	93535060	rs180276
chr7	93536802	93536803	rs36038530
chr7	93538294	93538295	rs180273
chr8	117957300	117957301	rs921067
chr8	117960146	117960147	rs2921722
chr8	117960213	117960214	rs3020107
chr8	117960385	117960386	rs3020108
chr8	117966293	117966294	rs3020110
chr8	117972027	117972028	rs1596895
chr8	117972194	117972195	rs3020114
chr8	117973344	117973345	rs2921719
chr8	117974267	117974268	rs3020115
chr8	117975152	117975153	rs3020116
chr8	117978639	117978640	rs34247878
chr8	117990697	117990698	rs35071148
chr8	117992983	117992984	rs4510896
chr8	117995886	117995887	rs7842705
chr8	117997531	117997532	8:118066713
chr8	117997644	117997645	rs10099121
chr8	117999182	117999183	rs4242567
chr8	117999278	117999279	rs4242568
chr8	118000754	118000755	rs12542825
chr8	118001866	118001867	rs4269571
chr8	118002575	118002576	rs4132473
chr8	118002806	118002807	rs6469667
chr8	118005135	118005136	rs3019886
chr8	118005213	118005214	rs35929196

chr8	118006570	118006571	rs3020119
chr8	118008220	118008221	rs2938863
chr8	118010779	118010780	rs3020121
chr8	118011863	118011864	rs1505525
chr8	118012029	118012030	rs3020122
chr8	118012264	118012265	rs2938864
chr8	118013962	118013963	rs3019890
chr8	118014675	118014676	rs2047964
chr8	118015902	118015903	rs1995581
chr8	118016842	118016843	rs2047963
chr8	118017286	118017287	rs3019879
chr8	118019620	118019621	rs3019883
chr8	118020170	118020171	rs3019884
chr8	118021471	118021472	rs2938861
chr8	118021877	118021878	rs34103819
chr8	118025644	118025645	rs3019885
chr8	15472193	15472194	rs11781005
chr8	15479637	15479638	rs11782065
chr8	15479715	15479716	rs11785241
chr8	15481326	15481327	rs35141734
chr8	15481841	15481842	rs63366060
chr8	15482652	15482653	rs1835141
chr8	15482958	15482959	rs13257136
chr8	15484858	15484859	rs35422110
chr8	15485128	15485129	rs11774766
chr8	15487971	15487972	rs56403992
chr8	15488340	15488341	rs7017500
chr8	15490094	15490095	rs35246946
chr8	15490340	15490341	rs34768042
chr8	15492520	15492521	rs35920749
chr8	15494370	15494371	rs6530891
chr8	15500339	15500340	rs35440712
chr8	15500651	15500652	rs12541338
chr8	15500966	15500967	rs12541402
chr8	15503202	15503203	rs35445780
chr8	15503789	15503790	rs13248414
chr8	15503951	15503952	rs34160987
chr8	15506300	15506301	rs55713110
chr8	15508430	15508431	rs1975058
chr8	15508900	15508901	rs11782230
chr8	15509104	15509105	rs11777115
chr8	15509604	15509605	rs62502091
chr8	15509766	15509767	rs62502092
chr8	15510890	15510891	rs35885223
chr8	15513204	15513205	rs10100681
chr8	15514593	15514594	rs62502094
chr8	15515256	15515257	rs13278140

chr8	15517714	15517715	rs13264325
chr8	15518410	15518411	rs60971907
chr8	15519206	15519207	rs1560218
chr8	15519231	15519232	rs1560217
chr8	15520415	15520416	rs35854756
chr8	15523242	15523243	rs2562743
chr8	15524406	15524407	rs66892516
chr8	15527476	15527477	rs35147004
chr8	15527983	15527984	rs354517
chr8	15529889	15529890	rs354515
chr8	15530309	15530310	rs11774277
chr8	15530341	15530342	rs11776648
chr8	15530767	15530768	rs13279670
chr8	15530768	15530769	rs7828245
chr8	15531505	15531506	8:15575877
chr8	15531831	15531832	rs35181248
chr8	15532451	15532452	rs6994908
chr8	15532567	15532568	rs4831759
chr8	15532584	15532585	rs4831760
chr8	15532825	15532826	rs1835140
chr8	15532879	15532880	rs1835139
chr8	15532920	15532921	rs5889591
chr8	15533139	15533140	rs1835138
chr8	15534248	15534249	rs11780499
chr8	15534443	15534444	rs354511
chr8	15535592	15535593	rs7819021
chr8	15535597	15535598	rs62502114
chr8	15535715	15535716	rs7818067
chr8	15536077	15536078	rs7844322
chr8	15537607	15537608	rs34530180
chr8	15537674	15537675	rs13276710
chr8	15537824	15537825	rs13277232
chr8	15537832	15537833	rs35831716
chr8	15537995	15537996	rs34672838
chr8	15540112	15540113	rs1961471
chr8	15540362	15540363	rs2098507
chr8	15540780	15540781	rs3940343
chr8	15541175	15541176	rs4298487
chr8	15541329	15541330	rs4319097
chr8	15542784	15542785	rs35103268
chr8	15543652	15543653	rs13250624
chr8	15543895	15543896	rs13251487
chr8	15543920	15543921	rs13251503
chr8	15544871	15544872	rs34599046
chr8	15545455	15545456	rs34747263
chr8	15545858	15545859	rs62502123
chr8	15546514	15546515	rs35772780

chr8	15546934	15546935	rs34918436
chr8	15546938	15546939	rs36061298
chr8	15546966	15546967	rs36111860
chr8	15547012	15547013	rs35174544
chr8	15547678	15547679	rs34974267
chr8	15549613	15549614	rs62502129
chr8	15550060	15550061	rs62502157
chr8	15550182	15550183	rs13266004
chr8	15550550	15550551	rs13265117
chr8	15550857	15550858	rs11785917
chr8	15550868	15550869	rs11782814
chr8	15550942	15550943	rs11785945
chr8	15551414	15551415	rs4831346
chr8	15551900	15551901	rs62502158
chr8	15554013	15554014	rs13271388
chr8	34236707	34236708	rs1870388
chr8	34325316	34325317	rs72634682
chr8	34325701	34325702	rs16882264
chr8	34327789	34327790	rs16882274
chr8	34328597	34328598	rs6995878
chr8	34329356	34329357	rs61643699
chr8	34331219	34331220	rs16882295
chr8	34331648	34331649	rs12546511
chr8	34336503	34336504	rs58413292
chr8	34343353	34343354	rs28540946
chr8	34343913	34343914	rs28366982
chr8	34345754	34345755	rs55752257
chr8	34347847	34347848	rs34289127
chr8	34348495	34348496	rs16882360
chr8	34351135	34351136	rs7012499
chr8	34352147	34352148	rs7827467
chr8	34352644	34352645	rs6468262
chr8	34353745	34353746	rs4739572
chr8	34354025	34354026	rs12375320
chr8	34354973	34354974	rs12547877
chr8	34357600	34357601	rs67405485
chr8	34361502	34361503	rs12156325
chr8	34362657	34362658	rs12681740
chr8	34364806	34364807	rs55733001
chr8	34368863	34368864	rs6983519
chr8	34389996	34389997	rs1106004
chr8	34392715	34392716	rs58740934
chr8	34393791	34393792	rs58415208
chr8	34396907	34396908	rs115635704
chr8	34404306	34404307	rs6984443
chr8	34407767	34407768	rs4739583
chr8	34411190	34411191	rs56819505

chr8	34414241	34414242	rs56091526
chr8	34414822	34414823	rs4260892
chr8	34415267	34415268	rs55999916
chr8	34415527	34415528	rs4291266
chr8	34416050	34416051	rs56251523
chr8	34416743	34416744	rs4472512
chr8	34418059	34418060	rs58091692
chr8	34423766	34423767	rs12679837
chr8	34424949	34424950	rs4587331
chr8	34429002	34429003	rs72640929
chr8	34430719	34430720	rs72640930
chr8	34435657	34435658	rs12676938
chr8	34437623	34437624	rs56201185
chr8	34438867	34438868	rs883181
chr8	34440104	34440105	rs72640938
chr8	34441682	34441683	rs16882460
chr8	34451825	34451826	rs72640948
chr8	34453453	34453454	rs10954967
chr8	34453921	34453922	rs12156120
chr8	34453941	34453942	rs12156296
chr8	34455987	34455988	rs16882486
chr8	34463348	34463349	rs59219883
chr8	34463618	34463619	rs55976920
chr8	34463859	34463860	rs16882556
chr8	34469645	34469646	8:34589188
chr8	34470865	34470866	rs72640985
chr8	34470984	34470985	rs72640986
chr8	34476365	34476366	rs57425318
chr8	34476469	34476470	rs72640997
chr8	34477752	34477753	rs4284044
chr8	34486990	34486991	rs57694108
chr8	34487890	34487891	rs4480120
chr8	34489288	34489289	rs16882599
chr8	34491216	34491217	rs953272
chr8	34497019	34497020	rs4313154
chr8	34497128	34497129	rs4314651
chr8	34498385	34498386	rs16882612
chr8	34498729	34498730	rs4451305
chr8	34498967	34498968	rs16882614
chr8	34506797	34506798	rs10673798
chr8	34507028	34507029	rs7837337
chr8	34507250	34507251	rs72644261
chr8	34507320	34507321	rs72644263
chr8	34509993	34509994	rs72644265
chr8	34510073	34510074	rs72644266
chr8	34511223	34511224	rs4439121
chr8	34511227	34511228	rs4332131

chr8	34511427	34511428	rs56703575
chr8	34511722	34511723	rs16882633
chr8	34513064	34513065	rs58806020
chr8	34513664	34513665	rs16882643
chr8	34514333	34514334	rs16882649
chr8	34514590	34514591	rs34237510
chr8	34515267	34515268	rs16882656
chr8	34515716	34515717	rs16882660
chr8	34516641	34516642	rs4739598
chr8	34517267	34517268	rs16882673
chr8	34517862	34517863	rs72629506
chr8	34520014	34520015	rs4577953
chr8	34520183	34520184	rs4297034
chr8	34520585	34520586	rs4612337
chr8	34521281	34521282	rs12545278
chr8	34521322	34521323	rs12547489
chr8	34522545	34522546	rs12546023
chr8	34522656	34522657	rs35008961
chr8	34522665	34522666	rs12543513
chr8	34522716	34522717	rs12543518
chr8	34522866	34522867	rs12543568
chr8	34522958	34522959	rs59950658
chr8	34523029	34523030	rs16882701
chr8	34523094	34523095	rs28540657
chr8	34523223	34523224	rs10503965
chr8	34523312	34523313	rs16882703
chr8	34523559	34523560	rs4263763
chr8	34524002	34524003	rs6987004
chr8	34524020	34524021	rs7005465
chr8	3589414	3589415	rs73187242
chr8	3590609	3590610	rs17067216
chr8	3591276	3591277	rs73187246
chr8	3592995	3592996	rs73187251
chr8	3593641	3593642	rs60828349
chr8	3594905	3594906	rs2623702
chr8	3595124	3595125	rs73187261
chr8	3596138	3596139	rs73187266
chr8	3596615	3596616	rs73187267
chr8	41615137	41615138	rs7006290
chr8	41616477	41616478	rs11989912
chr8	41621627	41621628	rs13263917
chr8	41621902	41621903	rs11997827
chr8	61862785	61862786	rs11995686
chr8	61864852	61864853	rs10110018
chr8	61866324	61866325	rs28688155
chr8	61866350	61866351	rs28583647
chr8	61869648	61869649	rs12335258

chr8	61874497	61874498	rs10086776
chr8	61876043	61876044	rs10104895
chr8	61877401	61877402	rs72652592
chr8	73236809	73236810	rs7006742
chr8	98166912	98166913	rs1835740
chr8	98171187	98171188	rs13258763
chr8	98174902	98174903	rs1835741
chr9	113291560	113291561	rs34834254
chr9	113291835	113291836	rs7853091
chr9	113291856	113291857	rs7853098
chr9	113291887	113291888	rs34764782
chr9	113291941	113291942	rs7853220
chr9	113294561	113294562	rs1931314
chr9	113294796	113294797	rs10117891
chr9	113295373	113295374	rs7873791
chr9	113295423	113295424	rs7873899
chr9	113295490	113295491	rs10759438
chr9	113296011	113296012	rs7036152
chr9	113296045	113296046	rs4300064
chr9	113296398	113296399	rs4300065
chr9	113296868	113296869	rs10739306
chr9	113297090	113297091	rs10759439
chr9	113297927	113297928	rs4460458
chr9	113300205	113300206	rs10759440
chr9	113300834	113300835	rs1889321
chr9	113300954	113300955	rs4599884
chr9	113301673	113301674	rs7030906
chr9	113301863	113301864	rs7045746
chr9	113302015	113302016	rs7046027
chr9	113302358	113302359	rs10817035
chr9	113303410	113303411	rs7848209
chr9	113303647	113303648	rs12001322
chr9	113303684	113303685	rs12001338
chr9	113303810	113303811	rs12001328
chr9	122219554	122219555	rs58554876
chr9	122227702	122227703	rs12235956
chr9	122243801	122243802	rs58379881
chr9	122244895	122244896	rs16908421
chr9	122247195	122247196	rs10984552
chr9	122247520	122247521	rs10984553
chr9	122247658	122247659	rs10984554
chr9	122247682	122247683	rs10984555
chr9	122247733	122247734	rs10984556
chr9	122247802	122247803	rs12237206
chr9	122247926	122247927	rs12235333
chr9	122248624	122248625	rs80242897
chr9	122249066	122249067	rs12238498

chr9	122249534	122249535	rs917709
chr9	122249689	122249690	rs10984557
chr9	122249903	122249904	rs73540220
chr9	122250258	122250259	rs73540223
chr9	122250436	122250437	rs78544256
chr9	122250587	122250588	rs73540226
chr9	122250599	122250600	rs77882033
chr9	122251072	122251073	rs59720051
chr9	122251145	122251146	rs58163718
chr9	122251255	122251256	rs7019298
chr9	122251768	122251769	rs7039410
chr9	122252358	122252359	rs7023869
chr9	122252549	122252550	rs73540236
chr9	122252586	122252587	rs57411773
chr9	122252792	122252793	rs58300957
chr9	122252849	122252850	rs59676902
chr9	122253547	122253548	rs7031568
chr9	122253739	122253740	rs7032085
chr9	122253997	122253998	rs7032141
chr9	122254046	122254047	rs7032151
chr9	122254395	122254396	rs36110172
chr9	122254438	122254439	rs1331594
chr9	122254604	122254605	rs1331595
chr9	122255447	122255448	rs73540254
chr9	122255507	122255508	rs73540255
chr9	122256484	122256485	rs10491525
chr9	122256521	122256522	rs57572849
chr9	122256835	122256836	rs10984559
chr9	122258576	122258577	rs10984561
chr9	17487944	17487945	rs2383024
chr9	17487970	17487971	rs2891111
chr9	20096453	20096454	rs7856353
chr9	20097116	20097117	rs5025178
chr9	20097432	20097433	rs2151582
chr9	20098120	20098121	rs75663449
chr9	20098274	20098275	rs78849874
chr9	20098710	20098711	rs16937883
chr9	20099580	20099581	rs77647874
chr9	20100121	20100122	rs114803662
chr9	20100651	20100652	rs7866672
chr9	20100941	20100942	rs6475404
chr9	20101220	20101221	rs6475405
chr9	20101298	20101299	rs6475406
chr9	20101431	20101432	rs7043572
chr9	20101895	20101896	rs74639128
chr9	20102267	20102268	rs7859032
chr9	20102308	20102309	rs11422910

chr9	20102513	20102514	rs7859266
chr9	20109017	20109018	rs76297615
chr9	20110645	20110646	rs75394562
chr9	20111177	20111178	rs74652854
chr9	20111603	20111604	rs1413266
chr9	20112500	20112501	9:20102501
chr9	20113079	20113080	9:20103080
chr9	20113400	20113401	rs79416980
chr9	20114288	20114289	rs78461553
chr9	20115469	20115470	rs78301898
chr9	20115656	20115657	rs10511676
chr9	20116150	20116151	rs76715200
chr9	20116196	20116197	rs16937896
chr9	20116591	20116592	rs16937898
chr9	20116980	20116981	rs75256031
chr9	20117032	20117033	rs75207602
chr9	20117618	20117619	rs75490274
chr9	20117946	20117947	rs78757274
chr9	20118130	20118131	rs79310211
chr9	20118547	20118548	rs75764448
chr9	20119176	20119177	rs75663356
chr9	20119327	20119328	9:20109328
chr9	20120022	20120023	rs75474826
chr9	20120617	20120618	rs74943830
chr9	20120682	20120683	rs79833182
chr9	20120999	20121000	rs77430582
chr9	20121944	20121945	rs74700468
chr9	20121969	20121970	rs78856972
chr9	20123967	20123968	rs75254812
chr9	20124091	20124092	rs77828555
chr9	20125258	20125259	rs6475409
chr9	20125393	20125394	rs6475410
chr9	20125535	20125536	rs7861225
chr9	20126261	20126262	rs78624775
chr9	20126563	20126564	rs76667488
chr9	32431930	32431931	rs10970975
chr9	32432564	32432565	rs1556138
chr9	32432646	32432647	rs1467713
chr9	32432680	32432681	rs1467712
chr9	32433525	32433526	rs10970976
chr9	32435675	32435676	rs10758139
chr9	32435977	32435978	rs2068116
chr9	32436682	32436683	rs10813815
chr9	32437248	32437249	rs10813816
chr9	32442491	32442492	rs10511906
chr9	565916	565917	rs7027930
chr9	6172379	6172380	rs7032572

chr9	6175854	6175855	rs72699186
chr9	6176870	6176871	rs72699188
chr9	6185294	6185295	rs12349858
chr9	6190075	6190076	rs1342326
chr9	6192795	6192796	rs2095044
chr9	6193454	6193455	rs2381416
chr9	6197376	6197377	rs10975479
chr9	6197391	6197392	rs1888909
chr9	6201162	6201163	rs1929995
chr9	6209696	6209697	rs992969
chr9	6210098	6210099	rs3939286
chr9	6211812	6211813	rs72699191
chr9	6213147	6213148	rs928412
chr9	6213386	6213387	rs928413
chr9	6213467	6213468	rs7848215
chr9	82013518	82013519	rs35709306
chr9	82013915	82013916	rs35777231
chr9	82013927	82013928	rs35255890
chr9	82015013	82015014	9:81204834
chr9	82015429	82015430	rs35850334
chr9	82015732	82015733	rs17800992
chr9	82015764	82015765	rs17082349
chr9	82017039	82017040	9:81206860
chr9	82017053	82017054	rs67055148
chr9	82017238	82017239	rs7042430
chr9	82017479	82017480	rs7045698
chr9	82019522	82019523	rs13287322
chr9	82020517	82020518	rs34791035
chr9	82027170	82027171	rs1987186
chr9	82028696	82028697	rs35051503
chr9	82029555	82029556	rs11138197
chr9	82030691	82030692	rs34849353
chr9	82030960	82030961	rs34128325
chr9	82031330	82031331	rs10867380
chr9	82032096	82032097	rs11138198
chr9	82033331	82033332	rs12238261
chr9	82039164	82039165	rs2378382
chr9	82039361	82039362	rs2378383
chr9	82042517	82042518	rs34217047
chr9	82043419	82043420	rs71496261
chr9	82043425	82043426	rs71496262
chr9	82043534	82043535	rs71496263
chr9	82044241	82044242	rs11138205
chr9	82044820	82044821	rs17348778
chr9	82045543	82045544	rs7045997
chr9	82046047	82046048	rs11138209
chr9	82046185	82046186	rs11138210

chr9	82046450	82046451	rs13298282
chr9	82046970	82046971	rs1417084
chr9	82047373	82047374	rs7020659
chr9	82048008	82048009	rs16857
chr9	82048327	82048328	rs7025226
chr9	82048642	82048643	rs1934609
chr9	82048887	82048888	rs2226021
chr9	82049299	82049300	rs11138211
chr9	82049460	82049461	rs11138212
chr9	82051322	82051323	rs6559477
chr9	82051903	82051904	rs7027401
chr9	82051946	82051947	rs7041857
chr9	82052197	82052198	rs11138213
chr9	82052461	82052462	rs1340122
chr9	82053065	82053066	rs13295991
chr9	82059953	82059954	rs11138219
chr9	82060527	82060528	rs11138220
chr9	82061560	82061561	rs11138223
chr9	82063202	82063203	rs13302771
chr9	82063682	82063683	rs11138225
chr9	82064818	82064819	rs11138229
chr9	82065320	82065321	rs11138230
chr9	82066163	82066164	rs11138231
chr9	82066391	82066392	rs13291348
chr9	82068090	82068091	rs11138232
chr9	82068328	82068329	rs11138233
chr9	82069379	82069380	rs11138234
chr9	82069807	82069808	rs11138236
chr9	82069970	82069971	rs11138237
chr9	82070746	82070747	rs71496274
chr9	82070823	82070824	rs71496275
chr9	82071209	82071210	rs71496276
chr9	82071352	82071353	rs41340048
chr9	82072622	82072623	rs34274683
chr9	82074707	82074708	rs10491785
chr9	82074921	82074922	rs10491786
chr9	82075114	82075115	rs10491787
chr9	82076464	82076465	rs11138241
chr9	82077247	82077248	rs11138243
chr9	82077302	82077303	rs11138244
chr9	82077884	82077885	rs17427625
chr9	82078207	82078208	rs12238088
chr9	82078214	82078215	rs12235355
chr9	82078453	82078454	rs1015311
chr9	82078894	82078895	rs7028619
chr9	82078912	82078913	rs7044969
chr9	82079075	82079076	rs7029232

chr9	82080834	82080835	rs11138247
chr9	82080896	82080897	rs11138248
chr9	82081213	82081214	rs17349547
chr9	82081602	82081603	rs17082442
chr9	82081974	82081975	rs12237276
chr9	82082840	82082841	rs17082449
chr9	82082962	82082963	rs11138249
chr9	82083081	82083082	rs11138250
chr9	82093029	82093030	rs11138256
chr9	82096193	82096194	rs10491791
chr9	82098376	82098377	rs34670593
chr9	82098951	82098952	rs13286432
chr9	82099388	82099389	rs13286985
chr9	82099907	82099908	rs13291741
chr9	82103051	82103052	rs11138260
chr9	82104069	82104070	rs13296031
chr9	82104833	82104834	rs11138261
chr9	82109046	82109047	rs11138264
chr9	82115498	82115499	rs17350468
chr9	82116365	82116366	rs71496279
chr9	82117203	82117204	rs17350573

Table S3: Number of asthma and non-asthma SNPs in 51 predicted chromatin states by Ernst and Kellis, 2010.

ID	state	description	# asthma SNPs	# non-asthma SNPs	% asthma SNPs	% non-asthma SNPs	enrichment
1	Promoter states	Promoter Upstream States	6	13,099	0.24%	0.12%	2.06
2			7	16,795	0.28%	0.15%	1.88
3			5	19,698	0.20%	0.17%	1.14
4		Repressed Promoter	6	20,812	0.24%	0.18%	1.3
5		TSS states	6	12,058	0.24%	0.11%	2.24
6			9	13,932	0.36%	0.12%	2.91
7			3	9,765	0.12%	0.09%	1.38
8		Transcribed Promoter States	3	8,265	0.12%	0.07%	1.64
9			12	10,007	0.48%	0.09%	5.41
10			0	9,044	0.00%	0.08%	N/A
11			4	10,066	0.16%	0.09%	1.79
12	Transcribed states	Transcribed 5'proximal States	7	16,157	0.28%	0.14%	1.95
13			6	42,759	0.24%	0.38%	0.63
14			10	23,728	0.40%	0.21%	1.9
15			7	77,355	0.28%	0.68%	0.41
16			15	91,437	0.60%	0.81%	0.74
17		Transcribe less 5' proximal States	3	26,751	0.12%	0.24%	0.51
18			9	76,404	0.36%	0.68%	0.53
19			30	210,764	1.20%	1.86%	0.64
20		Candidate strong enhancer in transcribed regions	4	15,911	0.16%	0.14%	1.13
21		Spliced exons/GC Rich	33	25,445	1.31%	0.22%	5.85
22			9	55,146	0.36%	0.49%	0.74
23			9	88,593	0.36%	0.78%	0.46
24		Transcribed 5' Distal States	29	74,693	1.16%	0.66%	1.75
25			24	118,747	0.96%	1.05%	0.91
26			121	567,637	4.82%	5.02%	0.96
27		End of Transcription; exons; high expression	11	40,948	0.44%	0.36%	1.21
28		ZNF Genes; KAP1 repressed state	1	20,277	0.04%	0.18%	0.22
29	intergenic states	Candidate strong distal enhancer states	15	18,020	0.60%	0.16%	3.75
30			16	20,498	0.64%	0.18%	3.52
31		Intergenic H2AZ with open chromatin/TF binding; Candidate distal enhancer	2	20,644	0.08%	0.18%	0.44
32		Candidate weaker distal enhancer	7	31,207	0.28%	0.28%	1.01
33		Candidate distal enhancer	10	41,263	0.40%	0.36%	1.09
34		Proximal to active enhancers; Alu repeats	78	77,956	3.11%	0.69%	4.51
35		Active intergenic regions not enhancer specific	48	92,750	1.91%	0.82%	2.33

36	Active	Active intergenic further from enhancers; Alu repeats	163	482,754	6.49%	4.27%	1.52
37		Non-repressive intergenic domains; Alu repeats	430	1,317,552	17.13%	11.64%	1.47
38		H2AZ specific state	26	81,225	1.04%	0.72%	1.44
39		CTCF Island; Candidate Insulator	2	20,556	0.08%	0.18%	0.44
40	Repressed states	Unmappable	42	336,109	1.67%	2.97%	0.56
41		Heterochromatin; Nuclear Lamina; Most A/T rich	446	3,225,739	17.77%	28.51%	0.62
42		Heterochromatin; Nuclear Lamina; ERVL repeats	9	61,342	0.36%	0.54%	0.66
43		Heterochromatin	604	3,262,102	24.06%	28.83%	0.83
44		Heterochromatin; Nuclear Lamina; Less exon depleted	23	108,645	0.92%	0.96%	0.95
45		Specific Repression	54	154,302	2.15%	1.36%	1.58
46	Repetitive states	Simple repeats (CA)n, (TG)n	0	13,996	0.00%	0.12%	N/A
47		L1/LTR Repeats	133	113,064	5.30%	1.00%	5.3
48		Satellite Repeats	13	102,704	0.52%	0.91%	0.57
49			0	13,130	0.00%	0.12%	N/A
50			0	2,725	0.00%	0.02%	N/A
51			0	698	0.00%	0.01%	N/A

Table S4. Asthma SNPs enrichment calculated for four different GWA SNP panels.

Affymetrix 6.0

	asthma SNPs out of 254	non-asthma SNPs out of 909040	enrichment
CD4+ T cells	49	73,688	2.38
Liver	42	83,704	1.8
Adipose Nuclei	53	129,779	1.46
ADM stem cells	50	148,644	1.2
Kidney	24	72,207	1.19
Brain	33	115,953	1.02
Skeletal Muscle	34	98,926	1.23
Breast Myoepithelial cells	30	92,281	1.16

Illumina 550

	asthma SNPs out of 188	non-asthma SNPs out of 560784	enrichment
CD4+ T cells	34	50,449	2.01
Liver	33	55,689	1.77
Adipose Nuclei	44	86,020	1.53
ADM stem cells	45	94,779	1.42
Kidney	19	47,859	1.18
Brain	28	76,720	1.09
Skeletal Muscle	25	65,082	1.15
Breast Myoepithelial cells	26	59,677	1.3

Illumina 650

	asthma SNPs out of 216	SNPs out of 660172	enrichment
CD4+ T cells	38	58,176	2
Liver	40	64,437	1.9
Adipose Nuclei	47	99,823	1.44
ADM stem cells	49	110,698	1.35
Kidney	20	55,259	1.11
Brain	31	88,747	1.07
Skeletal Muscle	25	75,442	1.01
Breast Myoepithelial cells	30	69,186	1.33

Illumina 1MDuo

	asthma SNPs out of 376	SNPs out of 1203343	enrichment
CD4+ T cells	79	133,446	1.89
Liver	74	143,197	1.85
Adipose Nuclei	87	200,116	1.56
ADM stem cells	86	202,541	1.52
Kidney	42	114,259	1.32
Brain	56	175,978	1.14
Skeletal Muscle	50	157,120	1.14
Breast Myoepithelial cells	52	135,443	1.37

Table S5. Enrichment of asthma-SNPs in genomic regions in which there are tissue specific enhancers indicated by the column headers, and anywhere from 0 to seven additional cell types that also have an enhancer in that region indicated by the rows.

# of tissue specific enhancers	Adipose Nuclei	Skeletal Muscle	Liver	Kidney	Adipose stem (ADM) cells	CD4+ T cells	Breast Myoepithelial cells	Brain
1	1.02	0.28	1.83	0.7	1.08	3.26	0.4	0.34
2	1.22	0.32	1.39	0.64	1.07	2.82	0.42	0.43
3	1.27	0.38	1.39	0.72	1.11	2.84	0.48	0.42
4	1.18	0.45	1.29	0.71	1.09	2.57	0.55	0.43
5	1.14	0.57	1.24	0.78	1.1	2.37	0.57	0.52
6	1.18	0.67	1.31	0.9	1.12	2.32	0.69	0.61
7	1.19	0.75	1.29	0.98	1.13	2.21	0.75	0.69
8	1.21	0.83	1.31	1.06	1.15	2.11	0.84	0.77

Figure S1. Asthma-associated SNPs and H3K4me1 (enhancer) enriched regions in the human IKZF3 locus of different cell/tissue types.

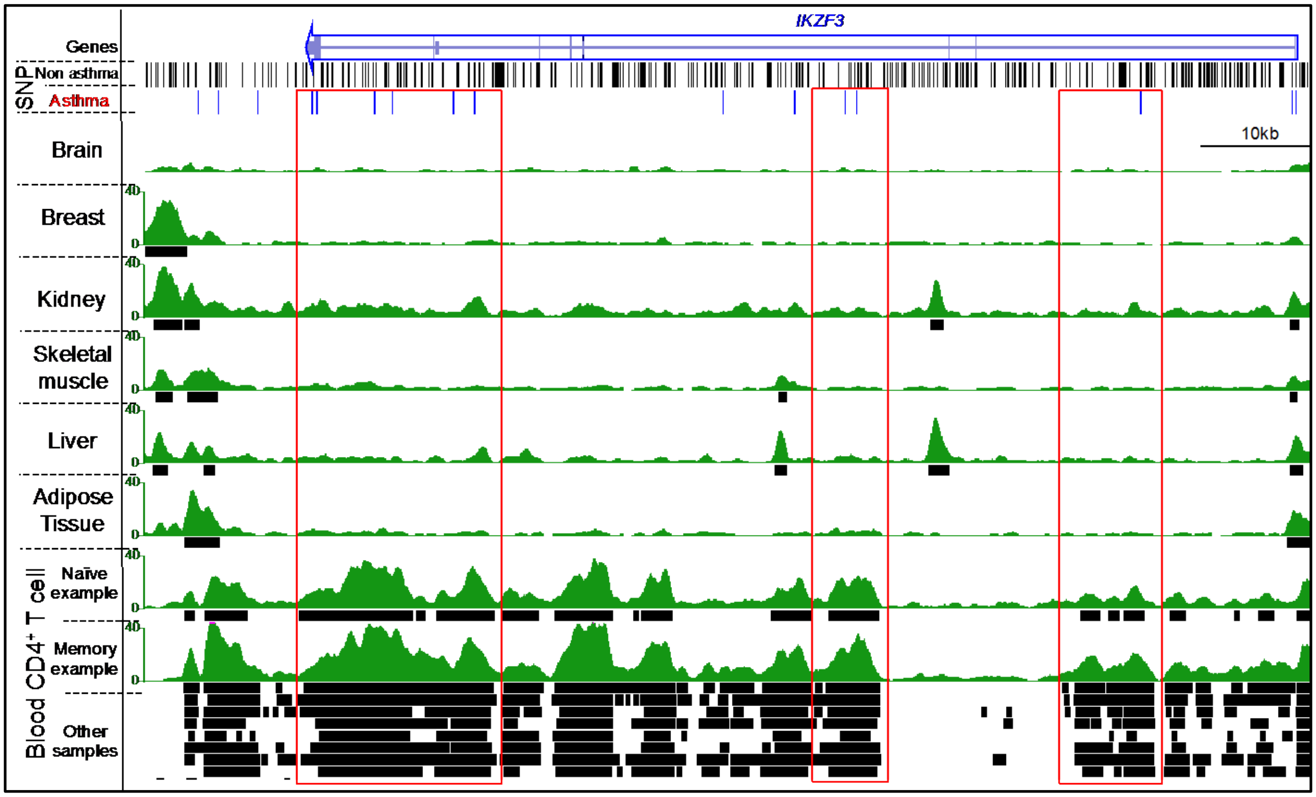

From top to bottom, using the UCSC genome browser, are displayed: the gene track (genes), all the SNPs not associated with asthma, the SNPs associated with asthma (red are GWAS-identified SNPs, blue are SNPs in linkage disequilibrium), H3K4me1 ChIP-seq track (green) for different cell/tissue types (named on the left) underlined by the corresponding peak-calling track (black boxes). For the blood CD4+ T cells, peak calling tracks from seven samples/cell-types are displayed. The red box shows an H3K4me1 peak that is present only in CD4+ T cells.
